# Human liver single nuclear RNA sequencing implicates BMPR2, GDF15, arginine, and estrogen in portopulmonary hypertension

**DOI:** 10.1038/s42003-023-05193-3

**Published:** 2023-08-09

**Authors:** Arun Jose, Jean M. Elwing, Steven M. Kawut, Michael W. Pauciulo, Kenneth E. Sherman, William C. Nichols, Michael B. Fallon, Francis X. McCormack

**Affiliations:** 1 Department of Medicine, University of Cincinnati College of Medicine, Cincinnati, OH USA; 2 Department of Medicine, Perelman School at the University of Pennsylvania, Philadelphia, PA USA; 3 Division of Human Genetics, Cincinnati Children’s Hospital Medical Center and Department of Pediatrics, University of Cincinnati College of Medicine, Cincinnati, OH USA; 4 Department of Medicine, University of Arizona, Phoenix, AZ USA

**Keywords:** Transcriptomics, Vascular diseases

## Abstract

Portopulmonary hypertension (PoPH) is a type of pulmonary vascular disease due to portal hypertension that exhibits high morbidity and mortality. The mechanisms driving disease are unknown, and transcriptional characteristics unique to the PoPH liver remain unexplored. Here, we apply single nuclear RNA sequencing to compare cirrhotic livers from patients with and without PoPH. We identify characteristics unique to PoPH in cells surrounding the central hepatic vein, including increased growth differentiation factor signaling, enrichment of the arginine biosynthesis pathway, and differential expression of the bone morphogenic protein type II receptor and estrogen receptor type I genes. These results provide insight into the transcriptomic characteristics of the PoPH liver and mechanisms by which PoPH cellular dysfunction might contribute to pulmonary vascular remodeling.

## Abstract (web summary)

Single-nucleus RNA-sequencing is used to investigate cirrhotic liver with PoPH and identifies diminished circulating BMP9, excess estrogen levels, altered arginine metabolism, and dysregulated GDF15 signaling may contribute to pulmonary vascular remodeling in PoPH

## Introduction

Pulmonary arterial hypertension (PAH) encompasses a diverse group of vascular diseases that lead to progressive right heart failure and death^1,2^. Portopulmonary hypertension (PoPH) is a particularly lethal subtype of PAH occurring in the setting of portal hypertension, associated with a 5-year mortality in excess of 60%, that is believed to afflict up to 6% of all chronic liver disease patients^3–6^. PoPH is known to exhibit pulmonary arterial vasculopathy and hemodynamic characteristics that are similar to other forms of PAH. Clinical studies comparing PoPH to non-PoPH portal hypertensive liver disease have previously implicated a number of potential biomarkers and genetic risk factors that may be involved in PoPH pathogenesis, including a deficiency in circulating bone morphogenic protein type 9 (BMP9) and genetic variation in estrogen signaling and metabolism^4,7–11^. To date, the biological and transcriptional characteristics unique to the PoPH liver have yet to be explored, and the mechanisms linking portal hypertension to pulmonary vascular remodeling in PoPH remain poorly defined.

Single nucleus RNA sequencing (snRNAseq) provides detailed gene expression profiles of individual cellular populations from dissociated segments of whole tissue. This technology has provided novel insights into the functions and interactions of resident cell populations in human tissue in health and disease, including PAH and liver cirrhosis^12–15^. Despite the widespread adoption of this platform to provide biological information about human pulmonary vascular disease at the resolution of a single cell, to our knowledge single cell technology has never been applied to the study of pulmonary vascular disease due to liver disease.

We anticipated that the transcriptomic profiles of PoPH human liver tissue cells would differ from non-PoPH human liver tissue derived from patients with cirrhosis, and would provide insight into the mechanisms of PoPH. We performed snRNAseq on human PoPH and non-PoPH cirrhosis liver tissue, using a combination of differential gene expression, pathway analysis, immunohistochemistry, and circulating biomarker assessments to identify and validate biological characteristics unique to PoPH.

## Results

### Liver snRNAseq identifies multiple distinct clusters

We applied snRNAseq to liver tissue samples from nine patients, acquired at the time of liver transplantation (Table 1). The majority of subjects were white females with alcoholic cirrhosis (*N* = 5, 56%). The subjects with PoPH (*N* = 4) had moderate to severe precapillary pulmonary vascular disease, with mean pulmonary arterial pressure (mPAP) ranging from 45 mmHg to 76 mmHg, and pulmonary vascular resistance (PVR) ranging from 4.8 Wood units to 16.1 Wood units, at the time of diagnosis.

**Table 1:** Demographic and clinical characteristics of subjects providing liver tissue.

Subjects providing liver tissue for snRNAseq (*N* = 9)														
Sample	Cirrhosis		Age		Race/Ethnicity		Sex		MELD	mPAP		PVR	PCWP	Rx
452 (control)	EtOH		48		B		M		36	–		–	–	–
458 (control)	HCV		60		W		M		10	–		–	–	–
479 (control)	EtOH		69		W		F		7	–		–	–	–
566 (control)	PBC		67		W		F		40	–		–	–	–
593 (control)	EtOH		34		W		M		25	–		–	–	–
397 (PoPH)	NASH		55		W		F		11	60		6.5	13	PDE-5i, ERA, oral prostanoid
455 (PoPH)	HCV		71		W		F		15	45		4.8	15	IV Epoprostenol, PDE-5i
448 (PoPH)	EtOH		64		W		F		7	50		8.2	14	PDE-5, ERA
557 (PoPH)	EtOH		42		W		F		7	76		16.1	13	IV Epoprostenol, PDE-5i

Subjects providing liver tissue for immunohistochemistry (*N* = 11)														
Sample	Age	Sex		Race/Ethnicity		MELD		Fibrosis Stage (Batts-Ludwig)			Disease			
POL1	71	F		W		15		Stage 2–3			PoPH			
POL3	55	F		W		11		Stage 2			PoPH			
POL4	64	F		W		7		Stage 1			PoPH			
NPOL1	48	M		B		36		Stage 2			Non-PoPH cirrhosis			
NPOL3	70	M		W		32		Stage 1			Non-PoPH cirrhosis			
NPOL5	64	M		W		18		Stage 1			Non-PoPH cirrhosis			
NPOL6	69	F		W		7		Stage 2			Non-PoPH cirrhosis			
NPOL7	45	M		W		11		Stage 4			Non-PoPH cirrhosis			
NL2	18	M		B		–		–			Healthy liver removed for trauma			
NL3	23	F		B		–		–			Healthy liver removed for ruptured adenoma			
NL4	58	F		W		–		–			Healthy liver removed for abdominal bleeding			

*B* black, *W* white, *F* female sex, *M* male sex, *EtOH* alcoholic, *HCV* hepatitis C virus, *PBC* primary biliary cirrhosis, *NASH* non-alcoholic steatohepatitis, *mPAP* mean pulmonary arterial pressure in mmHg, *PVR* pulmonary vascular resistance in Wood units, *PCWP* pulmonary capillary wedge pressure in mmHg, *Rx* targeted PAH therapy at time of liver transplantation, *PDE-5i* phosphodiesterase 5 inhibitor, *ERA* endothelin receptor antagonist.

**Fig. 1:**
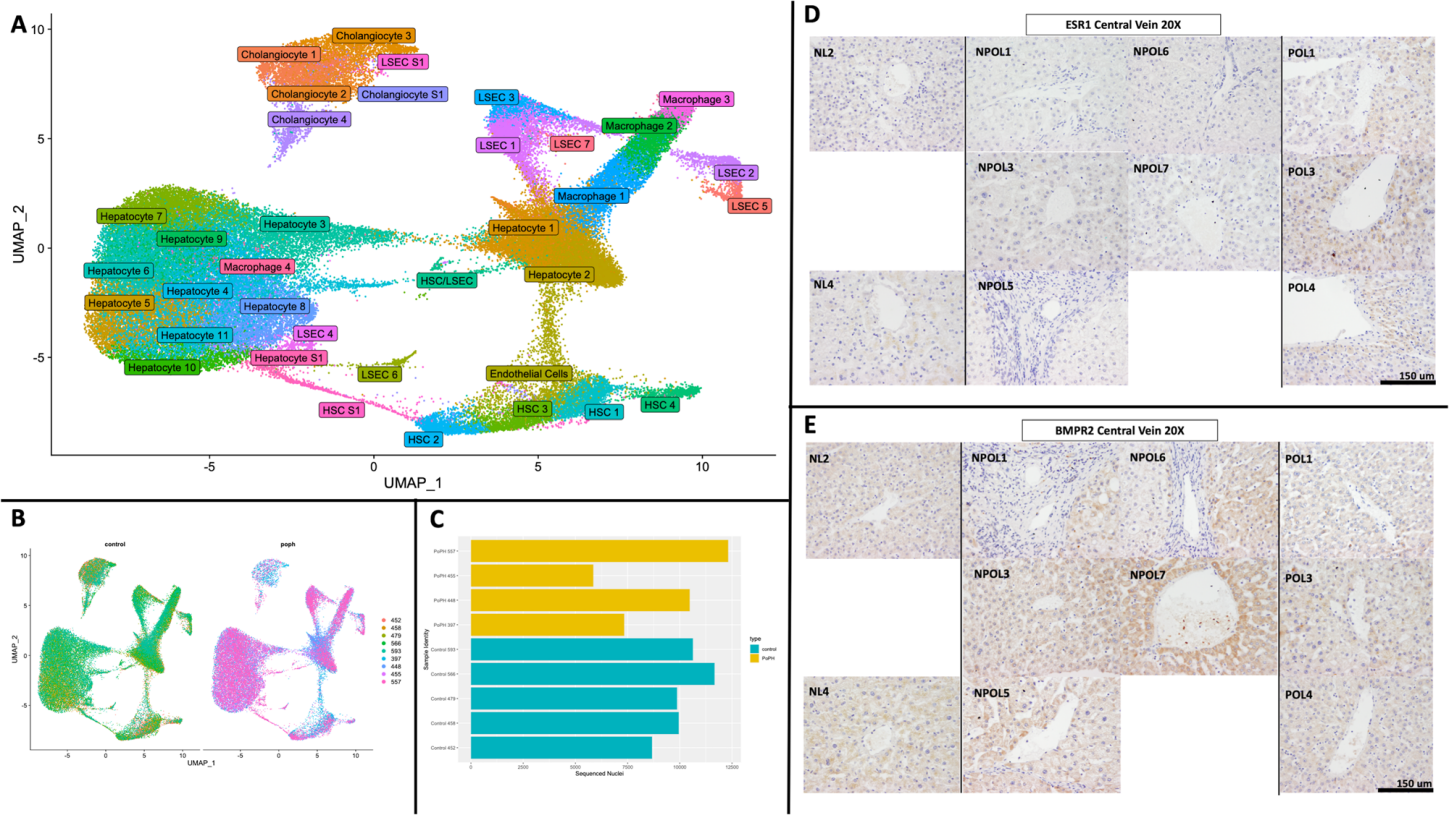
UMAP Projection of PoPH and non-PoPH cirrhosis tissue and immunohistochemistry of human liver tissue samples. UMAP projection of nuclei derived from PoPH and non-PoPH cirrhosis tissue (**A**), with contribution of each tissue sample to all identified clusters (**B**, **C**). Dot Plot showing differential gene expression of arginine biosynthesis pathway genes (*ASL, GPT2, ARG1, GOT1, ASS1, CPS1*) in peri-central PoPH clusters relative to non-PoPH cirrhosis clusters. Increased expression of *ESR1* (**D**) and decreased expression of *BMPR2* (**E**) are noted in the region surrounding the central vein in PoPH (right-most column, POL1-POL3) as compared to non-PoPH cirrhosis (middle column, NPOL1–NPOL7). This pattern of protein expression corresponds to the pattern of differential gene expression of *BMPR2* and *ESR1* in PoPH clusters relative to non-PoPH cirrhosis clusters. Normal healthy liver (NL2, NL4) is also presented as an additional comparator. Note that limited tissue samples prevented us from testing BMPR2 and ESR1 in NL3 samples.

After processing and quality control, a total of 81,118 unique nuclei remained for snRNAseq analysis. Following integration, clustering, sub-clustering, and dimensional reduction, a total of 36 distinct cellular clusters were identified (Fig. 1A). The library size (number of UMIs detected per cell) ranged from 1680 to 4884, and the number of genes detected per cell ranged from 1226 to 2289. Although more non-PoPH cirrhosis liver nuclei (*N* = 49,339, 60.8%) contributed to the full dataset relative to PoPH liver nuclei (*N* = 31,779, 39.2%), all samples contributed nuclei to all clusters for both PoPH and non-PoPH cirrhosis (Fig. 1B, C). In the final integrated dataset several major liver cell types were well represented (named based on their differentially expressed marker genes), including multiple distinct clusters of hepatocytes, cholangiocytes, macrophages, liver sinusoidal endothelial (LSEC), and hepatic stellate (HSC) cells (Supplementary Figs. 1, 2).

### PoPH nuclei differentially express genes related to BMPR2 and estrogen signaling

Differentially expressed genes for each cluster were generated from the full dataset (Supplementary Data 1). We first examined the differential expression of genes previously linked to PoPH in our dataset, either through the bone morphogenic protein system (*BMPR2, GDF2, BMP10, ENG, ACVRL1*)^9,10^ or the estrogen signaling pathway (*CYP19A1, ESR1, CYP1B1*)^7,8^. We identified significant downregulation of *BMPR2* expression across multiple PoPH hepatocyte clusters, and significant upregulation of *ESR1* across PoPH hepatocyte, cholangiocyte, and macrophage clusters (Supplementary Table 1). We observed differential expression of sex-specific genes (*XIST, UTY, TTTY14*) across multiple clusters, but this pattern aligned with the overrepresentation of female sex in PoPH compared to the non-PoPH cohort (Supplementary Fig. 3). When controlling for subject sex, differential gene expression of *ESR1* remained significantly higher in PoPH female hepatocyte clusters relative to non-PoPH cirrhosis females (Supplementary Fig. 4, Supplementary Table 1). We did not observe differential expression of the *GDF2* gene (encoding the BMP9 protein) in our dataset, and although multiple HSC and LSEC PoPH clusters differentially expressed the *ENG* gene (encoding endoglin, the soluble ligand trap for BMP9), this pattern of *ENG* expression did not remain significant after false discovery rate correction^9,16,17^.

### PoPH nuclei differential gene expression predict enrichment for pathways involved in fluid shear stress, estrogen signaling, and apelin signaling

Turning our attention to pathway enrichment analysis for the full dataset, we first examined upregulated pathways which involved the above significant differentially expressed genes, *BMPR2* and *ESR1* (specifically KEGG pathways for estrogen signaling, fluid shear stress and atherosclerosis, hippo signaling, and TGF-beta signaling) (Supplementary Table 1). We observed significant predicted enrichment for the fluid shear stress and atherosclerosis pathway (hsa05418) across multiple PoPH clusters, with enrichment of estrogen signaling (hsa04915), Hippo signaling (hsa04390), and transforming growth factor beta signaling (hsa04350) also predicted but to a lesser extent. Notably, we observed significant predicted enrichment for the apelin signaling pathway (hsa04371), which has been implicated together with estrogen and BMP9 as a mediator of *BMPR2* expression in pulmonary hypertension^18,19^.

### PoPH nuclei differentially express genes for inflammation

PAH is a disease that has been strongly linked to inflammation, and levels of different inflammatory cytokines and chemokines have shown promise as PAH-specific biomarkers with prognostic value^20,21^. Given this association, we then examined our snRNAseq dataset for a PoPH-specific inflammatory signature. Although a number of inflammatory cytokines and chemokines were differentially expressed in PoPH nuclei as compared to non-PoPH cirrhosis nuclei across multiple clusters, the strongest associations were for increased *IL6R* and *IL4R* expression and decreased *IL32* expression in PoPH clusters (Supplementary Table 2). Decreased *IL34* expression was also observed, but this pattern was limited to PoPH HSC clusters. PoPH macrophage clusters also demonstrated a unique signature, with increased *CXCL2* and *CD163* expression, and diminished *MIF* expression, relative to non-PoPH cirrhosis macrophage clusters (Supplementary Fig. 5).

### PoPH nuclei differentially express genes involved in the VEGF signaling pathway

Examining the results of our functional enrichment analysis, we identified pathways strongly implicated in the pathogenesis of pulmonary vascular disease (Vascular Endothelial Growth Factor (VEGF) signaling and HIF-1 Signaling)^22–25^. Subsequently, based on differential gene expression in both HSC and endothelial clusters, previous utility as a biomarker in PAH, and participation in the upregulated VEGF and HIF-1 signaling pathways noted above, we selected a member of the VEGF signaling cascade (Vascular Endothelial Growth Factor Receptor 1, FLT1) as a promising candidate biomarker for further validation^26,27^ (Supplementary Table 1). Unfortunately, given limited resource and sample availability, we were limited to a single promising candidate biomarker (soluble FLT1) for further validation.

### Human liver tissue protein expression supports differential gene expression of *BMPR2, ESR1*, and *FLT1* in PoPH liver

As PoPH nuclei demonstrated a pattern of differential gene expression involving the *BMPR2, ESR1*, and *FLT1* genes, we then sought to validate this pattern of gene expression using immunohistochemistry staining of human liver tissue from PoPH, non-PoPH cirrhosis, and normal liver tissue samples. PoPH samples demonstrated relatively more intense staining for both the ESR1 and FLT1 proteins (Fig. 1D, Supplementary Fig. 6), and relatively less staining for the BMPR2 protein (Fig. 1E), in the region surrounding liver central veins (the “peri-central” zone) relative to non-PoPH cirrhosis tissue. No differences in protein staining in the region surrounding the liver portal triad structure (the “peri-portal” zone) were readily apparent (Supplementary Fig. 6). Semi-quantitative immunohistochemistry measurements agreed with these qualitative observations, with PoPH peri-central regions demonstrating higher ESR1 and FLT1 protein staining, and lower BMPR2 protein staining, relative to non-PoPH cirrhosis, but no difference in staining of any protein across the peri-portal region (Supplementary Figure 7, Supplementary Table 3).

### PoPH peri-central cell network analysis predicts upregulation of the arginine biosynthetic pathway

**Fig. 2:**
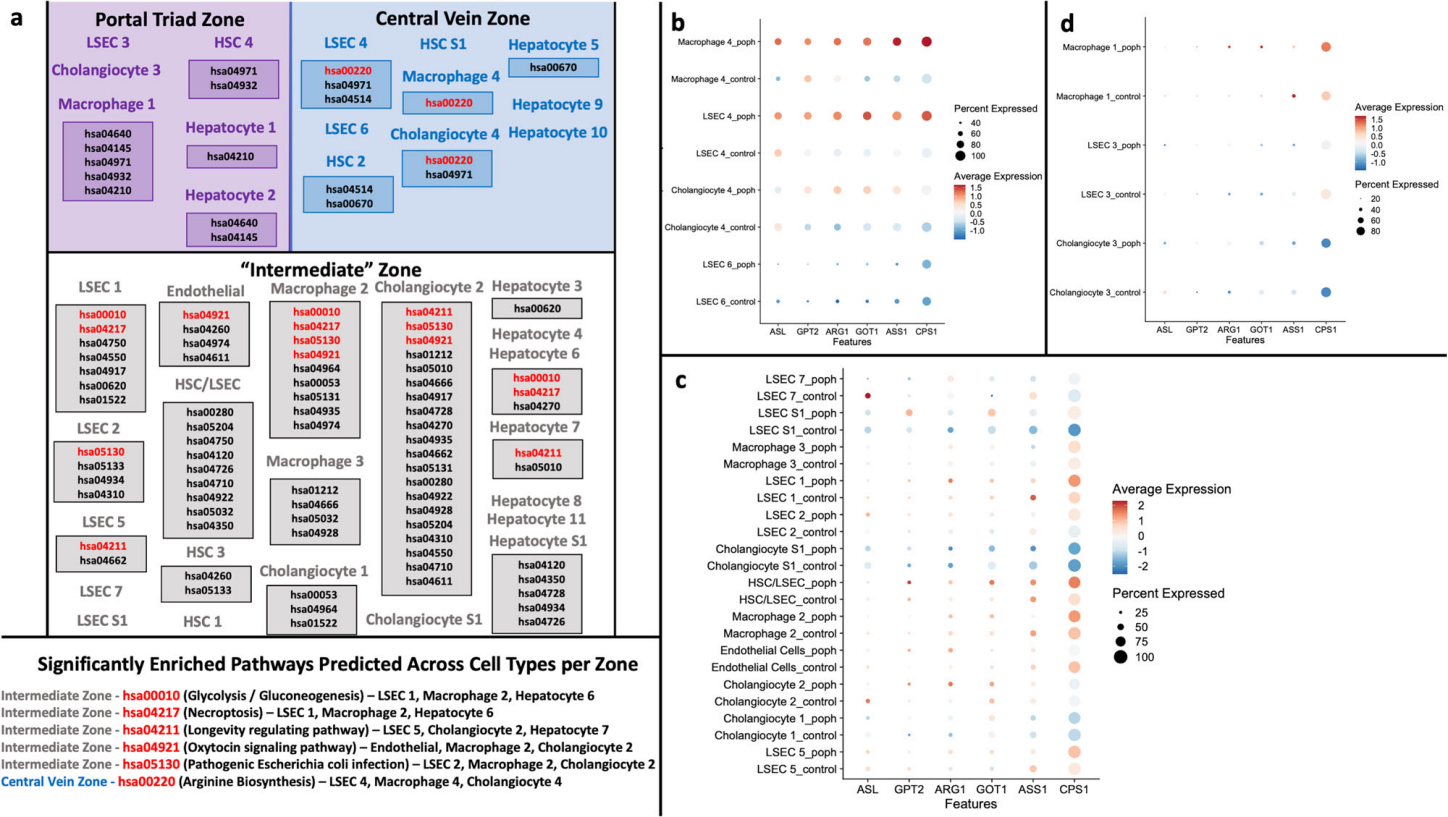
Pathway analysis results. Schematic of Cytoscape pathway analysis of PoPH clusters based on differential gene expression (**a**). First pathway analysis was used to identify significantly predicted signaling pathways (permutation-based test false discovery rate corrected *p* < 0.05) unique for a given cluster as compared to all other clusters of that cell type (i.e., LSEC 1, 2, etc. versus all other LSEC clusters). Then enriched pathways that were predicted in at least two different cell types (i.e., LSEC and Cholangiocytes) for a given liver zone (peri-portal, intermediate, and peri-central) were listed by cluster identity (bold KEGG pathway terms in boxes under cluster names). Significantly enriched pathways predicted across multiple (three or more) cell types for a given zone are highlighted in red, and described in greater detail under the schematic. Dot Plot demonstrating that differential expression of arginine biosynthesis pathway genes is unique to PoPH peri-central clusters (**b**), and mostly absent from intermediate zone clusters (**c**), and peri-portal clusters (**d**).

Given this spatially distinct pattern of liver *BMPR2* and *ESR1* expression, we next sought to identify other characteristics unique to PoPH cells likely to reside in the region surrounding the liver central vein (“peri-central” zone). We first grouped PoPH cell clusters into zones by using differential expression of known marker genes for the peri-central (*RSPO3, LGR5, FABP4, NOTUM, TBX3, GLUL, G6PC, GHR, CYP2E1,* and *ZNRF3* genes) and peri-portal (*ALB, EFNB2, MSR1, NTN4, JAG1, EPHX1*, and *EPCAM* genes) zones of the human liver^13,28–32^. Using this approach, we identified the Hepatocyte 1, Hepatocyte 2, HSC 4, LSEC 3, Cholangiocyte 3, and Macrophage 1 clusters as likely to be peri-portal (Supplementary Fig. 8), and the Hepatocyte 5, Hepatocyte 9, Hepatocyte 10, HSC 2, HSC S1, LSEC 4, LSEC 6, Macrophage 4, and Cholangiocyte 4 clusters as likely to be peri-central (Supplementary Fig. 9). The remaining clusters (Hepatocyte 3, Hepatocyte 4, Hepatocyte 6, Hepatocyte 7, Hepatocyte 8, Hepatocyte 11, Hepatocyte S1, HSC 1, HSC 3, LSEC 1, LSEC 2, LSEC 5, LSEC 7, LSEC S1, HSC/LSEC, Endothelial Cells, Cholangiocyte 1, Cholangiocyte 2, Cholangiocyte S1, Macrophage 2, Macrophage 3), which did not clearly express either a peri-central or a peri-portal pattern, were labeled “intermediate” zone clusters, likely residing between the central vein and portal triad of the liver (Supplementary Fig. 10, Supplementary Fig. 11). We then used the Cytoscape platform to construct interaction networks for each cluster, comparing signaling pathways that were differentially represented in a given cluster relative to all other clusters of that cell type (i.e., LSEC 1 versus all other LSEC clusters, LSEC 2 versus all other LSEC clusters, and so on) to identify pathways unique to a given cluster. For example, the LSEC 4 cluster was uniquely enriched for pathways including hsa00220 (arginine biosynthesis), hsa049217 (necroptosis), and hsa04514 (cell adhesion molecules), which were not significantly enriched by pathway analysis in any other LSEC cluster in our dataset (Fig. 2a). Grouping these unique pathways by zonal region (peri-central, intermediate, peri-portal), we were then able to describe zone-specific pathways uniquely enriched across multiple different cell types (for example, the hsa00220 pathway was enriched across the LSEC 4, Macrophage 4, and Cholangiocyte 4 clusters in the peri-central zone). Using this approach, we identified the arginine biosynthesis pathway (KEGG hsa00220) as the only predicted pathway uniquely and significantly enriched across several different PoPH peri-central cell clusters (LSEC 4, Cholangiocyte 4, Macrophage 4). These PoPH LSEC, Macrophage, and Cholangiocyte peri-central clusters also differentially expressed genes involved in the arginine biosynthesis pathway itself (*ASL, GPT2, ARG1, GOT1, ASS1, CPS1*) relative to non-PoPH cirrhosis (Fig. 2b), a pattern of gene expression absent from PoPH LSEC, Macrophage, and Cholangiocyte clusters in the intermediate or peri-portal zones (Fig. 2c, d).

### PoPH peri-central cells are uniquely enriched for growth differentiation factor 15 (GDF15) pathway signaling

**Fig. 3:**
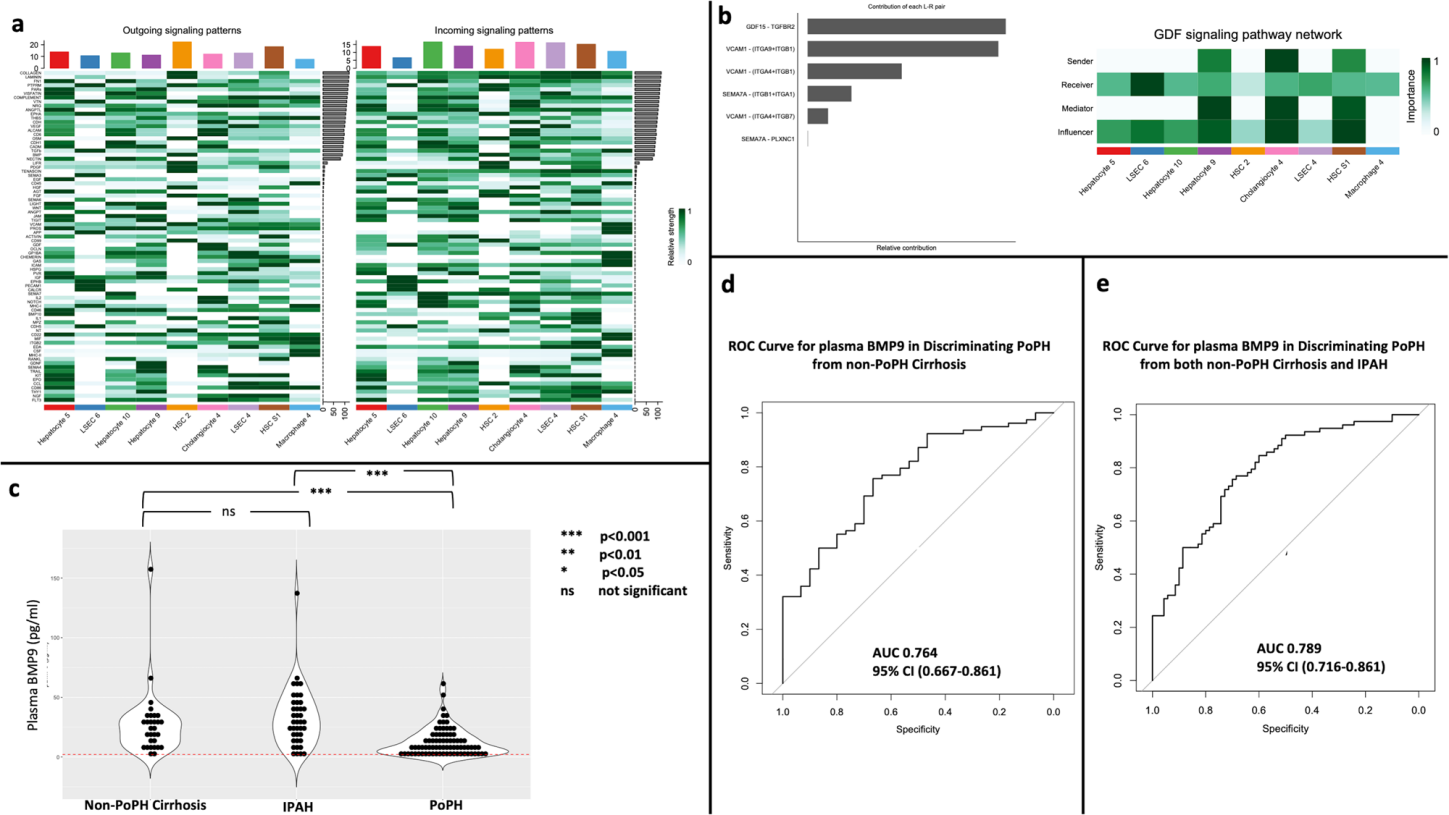
Cell communication networks and biomarker analysis results. Heatmaps showing predicted communication networks for PoPH liver peri-central clusters (**a**), ordered by total relative strength of gene expression (fold-change in gene expression colored in green) across all clusters on the y-axis, with given clusters on the x-axis. Comparing predicted communication for PoPH peri-central clusters, PoPH peri-portal clusters, and non-PoPH cirrhosis peri-central clusters, signaling through the GDF, VCAM, and SEMA7A pathways were the only predicted pathways unique to PoPH peri-central clusters (**b**, left). Relative contribution refers to the ratio of how much a given receptor-ligand pair contributes to the full communication network (defined by GDF, VCAM, and SEMA7A signaling). Sending of the signal (secretion of GDF15) and mediation/influencing was most strongly predicted in the peri-central Hepatocyte 9, Cholangiocyte 4, and HSC S1 clusters (**b**, right). Significantly (Kruskal–Wallis test *p* < 0.05) lower levels of circulating plasma BMP9 are seen in PoPH samples relative to both non-PoPH cirrhosis and IPAH in the initial biorepository cohort (**c**), there is no significant difference in plasma BMP9 levels between IPAH and non-PoPH cirrhosis groups. The dashed red line represents the lower limit of detection of the plasma BMP9 assay. As demonstrated by ROC Curves, multivariable regression analysis confirms plasma BMP9 discriminates PoPH from non-PoPH cirrhosis (**d**) and PoPH from a mixed cohort of non-PoPH cirrhosis and IPAH subjects (**e**) in the initial biorepository cohort.

Using the *CellChat* platform, we then investigated how predicted communication networks for PoPH cells differed across zones (peri-central, peri-portal). We first constructed *CellChat* predicted signaling networks for the PoPH peri-central clusters, PoPH peri-portal clusters, and non-PoPH cirrhosis peri-central clusters. Focusing on the PoPH peri-central clusters (Fig. 3a), we then identified signaling pathways (GDF, VCAM, and SEMA7 signaling) uniquely enriched in PoPH peri-central clusters and not present in the other two regions (PoPH peri-portal clusters, non-PoPH cirrhosis peri-central clusters). These pathways were then ranked by their contribution to signaling pathways unique to PoPH peri-central clusters, and over-expression and release of GDF15 as a signaling ligand (KEGG pathway hsa04350) was identified as the pathway that contributed the most to unique signaling in PoPH peri-central clusters (Fig. 3b). The corresponding differential expression of the *GDF15* gene was only observed in a specific PoPH peri-central cluster (Hepatocyte 9) and the intermediate zone cluster Hepatocyte S1 (Supplementary Table 1).

### Circulating levels of plasma BMP9, but not FLT1, discriminate PoPH from non-PoPH Cirrhosis and IPAH

Noting the absence of differential gene expression for the BMP9 gene (*GDF2*) in PoPH clusters, we then sought to validate and confirm low circulating BMP9 levels as characteristic of PoPH in subjects with underlying liver cirrhosis, and to assess a possible discrepancy between BMP9 liver transcriptomic signature and circulating protein levels, which may have implications for PoPH disease pathogenesis^9–11^. Given differential expression across multiple PoPH clusters, we also examined circulating FLT1 as a candidate biomarker of PoPH. In the initial validation cohort, subjects across groups had a median age of 58, and the majority were White (Table 2). There was no significant difference between PoPH and non-PoPH cirrhosis patients in age, sex, or BMI at time of biobank enrollment. There were significantly more Black individuals in the non-PoPH cirrhosis cohort versus the PoPH cohort. Non-PoPH cirrhosis subjects had a median MELD score of 11, and almost half had cirrhosis secondary to the hepatitis C virus. In the second independent validation cohort from PVCLD2, the median age was 58 years, the majority of subjects were male (61%), and there were no significant differences between groups in age, sex, race/ethnicity, liver disease etiology and severity (MELD-Na score), or body surface area. PoPH subjects had moderate precapillary pulmonary vascular disease present (median mPAP 44 mmHg, median PVR 5.3 Wood units) at the time of sample acquisition.

**Table 2:** Demographics and clinical characteristics of initial and secondary validation cohorts.

Initial validation cohort			
Characteristic	PoPH (*N* = 80)	Non-PoPH cirrhosis (*N* = 50)	*p*-value
	Median (IQR) or frequency (%)	Median (IQR) or frequency (%)	
Age (years)	58 (53–63)	58 (54–63)	0.502
Female sex	37 (46.3%)	20 (40.0%)	0.605
Race/Ethnicity			0.004
White	73 (91.3%)	40 (80.0%)	
Black	3 (3.7%)	10 (20.0%)	
Other	4 (5.0%)	0 (0%)	
BMI (kg/m^2^)	29.6 (26.0–33.6)	29.0 (25.3–33.3)	0.680
MELD-Na Score	–	11 (7–13)	–
Etiology of liver disease			
EtOH	–	14 (28.0%)	–
HCV	–	23 (46.0%)	–
NASH	–	11 (22.0%)	–
Other/Mixed	–	2 (4.0%)	–
mPAP (mmHg)	50 (41–56)	–	–
CO (L/min)	5.1 (4.0–6.6)	–	–
CI (L/min/m^2^)	2.7 (2.2–3.3)	–	–
PVR (Wood units)	7.1 (4.7–9.9)	–	–
PVRI (Wood unit*m^2^)	13.1 (10.1–19.8)		
TAPSE on TTE (cm)	–	2.5 (2.1–2.7)	–
TTE TR jet (m/s)	–	2.3 (1.8–2.6)	–
RV/LV end-diastolic ratio on TTE	–	0.6 (0.5–0.7)	–

Second independent validation cohort			
Characteristic	PoPH (*N* = 25)	Non-PoPH cirrhosis (*N* = 75)	*p*-value
	Median (IQR) or frequency (%)	Median (IQR) or frequency (%)	
Age (years)	57 (55–63)	58 (53–63)	0.842
Female Sex	10 (40.0%)	29 (38.7%)	>0.99
Race/Ethnicity			0.711
White	22 (88.0%)	68 (90.7%)	
Black	2 (8.0%)	6 (7.9%)	
Other	1 (4.0%)	1 (1.3%)	
BSA (m^2^)	2.1 (2.0–2.2)	2.0 (1.9–2.2)	0.356
MELD-Na Score	15 (11–18)	14 (10–18)	0.657
Etiology of liver disease			0.875
EtOH	7 (28.0%)	18 (24.0%)	
EtOH/HCV	4 (16.0%)	11 (14.7%)	
HCV	3 (12.0%)	16 (21.3%)	
NASH	4 (16.0%)	12 (16.0%)	
PBC	3 (12.0%)	5 (6.7%)	
Other/Mixed	2 (8.0%)	8 (10.7%)	
mPAP (mmHg)	44 (40–51)	–	–
CO (L/min)	5.9 (4.6–7.4)	–	–
CI (L/min/m^2^)	3.0 (2.5–3.6)	–	–
PVR (Wood units)	5.3 (3.7–8.8)	–	–
PVRI (Wood unit*m^2^)	11.5 (8.9–16.4)	–	–

IPAH Cohort (*N* = 40)			
Characteristic		Median (IQR) or frequency (%)	
Age (years)		58 (53–65)	
Female Sex		20 (50.0%)	
Race/Ethnicity			
White		33 (82.5%)	
Black		5 (12.5%)	
Other		2 (5.0%)	
BMI (kg/m^2^)		30.7 (27.7–36.7)	
mPAP (mmHg)		50 (46–55)	
CO (L/min)		5.1 (4.0–5.8)	
CI (L/min/m^2^)		2.4 (2.2–2.9)	
PVR (Wood units)		7.9 (6.3–9.6)	
PVRI (Wood unit*m^2^)		15.7 (12.9–19.9)	

*p*-value obtained by Chi-squared test or Mann–Whitney-Wilcoxon test for categorical or continuous variables, respectively.

*PoPH* portopulmonary hypertension, *BMI* body mass index, *MELD* model for end-stage liver disease, *EtOH* alcoholic, *HCV* hepatitis C virus, *NASH* non-alcoholic steatohepatitis, *PBC* primary biliary cirrhosis, *mPAP* mean pulmonary arterial pressure, *CO* cardiac output, *CI* cardiac index, *PVR* pulmonary vascular resistance, *PVRI* pulmonary vascular resistance index, *TTE* transthoracic echocardiogram, *TR* tricuspid regurgitant, *RV* right ventricle, *LV* left ventricle, *BSA* body surface area, *IPAH* idiopathic pulmonary arterial hypertension.

In the initial validation cohort, plasma levels of FLT1 were significantly higher in PoPH subjects as compared to non-PoPH cirrhosis (Table 3). FLT1 levels discriminated PoPH from non-PoPH cirrhosis in the initial cohort (AUC of 0.817, 95% CI 0.727–0.907), even after adjusting regression models for several clinical covariates (age, sex, race/ethnicity, BMI). In the second independent validation cohort from PVCLD2, plasma FLT1 failed to retain discriminatory significance. There were no significant differences between PoPH and non-PoPH cirrhosis subjects in levels of serum NTproBNP in either cohort.

**Table 3:** Biomarker levels across validation cohorts and in regression models.

Initial discovery cohort—biomarker levels between groups									
Biomarker (pg/ml)	PoPH (*N* = 80)		Non-PoPH cirrhosis (*N* = 50)			IPAH (*N* = 40)			*p*-value
	Median (IQR)		Median (IQR)			Median (IQR)			
Plasma FLT1	310 (223–425)		153 (133–198)			300 (208–391)			<0.001
Serum NTproBNP	163 (128–249)		226 (135–295)			172 (118–250)			0.898
Plasma BMP9	9.3 (4.3–17.6)		23.2 (10.7–33.1)			29.6 (16.2–45.2)			<0.001

Second validation cohort—biomarker levels between groups									
Biomarker (pg/ml)		PoPH (*N* = 25)		Non-PoPH cirrhosis (*N* = 75)				*p*-value	
Plasma FLT1		192 (130–513)		175 (93–501)				0.519	
Serum NTproBNP		74 (60–107)		85 (59–165)				0.302	

Initial discovery cohort—logistic regression models									
Models with PoPH and Non-PoPH cirrhosis					Odds ratio		95% CI	*p*-value	
Univariable Plasma FLT1					12.04		4.31–41.18	<0.001	
Univariable Plasma BMP9					0.33		0.17–0.56	<0.001	
Univariable Serum NTproBNP					1.38		0.95–2.11	0.101	
Multivariable Plasma FLT1					11.91		3.86–47.28	<0.001	
Multivariable Plasma BMP9					0.34		0.16–0.61	0.001	

Second validation cohort—logistic regression models									
Models with PoPH and Non-PoPH cirrhosis					Odds ratio		95% CI	*p*-value	
Univariable Plasma FLT1					1.10		0.77–1.57	0.599	
Univariable Serum NTproBNP					0.57		0.21–1.30	0.212	

Initial discovery cohort—logistic regression models including IPAH subjects									
Model with PoPH, Non-PoPH cirrhosis, and IPAH					Odds ratio		95% CI	*p*-value	
Univariable Plasma BMP9					0.32		0.20–0.48	<0.001	
Multivariable Plasma BMP9					0.32		0.20–0.50	<0.001	

*p*-value obtained by Kruskal–Wallis test on log-transformed biomarker levels for comparisons between three groups (PoPH, non-PoPH Cirrhosis, IPAH), and by Mann–Whitney–Wilcoxon test on log-transformed biomarker levels for comparisons between two groups (PoPH, non-PoPH cirrhosis). Multivariable regression models are additionally adjusted for clinical covariates (age, sex, race, BMI).

*PoPH* portopulmonary hypertension, *FLT1* FMS related receptor tyrosine kinase 1, *BMP9* bone morphogenic protein type 9, *NTproBNP* N-terminal prohormone of brain natriuretic peptide.

Plasma levels of BMP9 were also significantly lower in PoPH subjects as compared to non-PoPH cirrhosis in the initial cohort, even after adjustment for age, sex, race/ethnicity, and BMI (logistic regression model AUC of 0.764, 95% CI 0.667–0.861) (Table 3, Fig. 3c, d). We then measured plasma BMP9 in the cohort of 40 IPAH subjects obtained from the PAH biobank, finding plasma BMP9 remained significantly lower in PoPH subjects when compared to those with IPAH (Table 3, Fig. 3c). We further validated that plasma BMP9 levels discriminated PoPH from IPAH subjects, maintaining discrimination for PoPH in the full cohort (PoPH, non-PoPH cirrhosis, and IPAH), even after adjustment for age, sex, race/ethnicity, and BMI (AUC of 0.789, 95% CI 0.716–0.861) (Fig. 3e, Table 3).

### Circulating levels of BMP9 and FLT1 fail to correlate with PoPH disease severity

There was no significant association between biomarker levels (plasma FLT1, serum NTproBNP, plasma BMP9) and hemodynamic disease severity (mPAP, PVR, cardiac index) in either cohort (Supplementary Table 4).

## Discussion

Here we report the use of snRNAseq sequencing to characterize differential gene expression in human liver tissue from subjects with PoPH. In this study, we observed differential gene expression of *BMPR2*, *ESR1*, and *FLT1* genes in livers from patients with PoPH relative to non-PoPH cirrhosis, patterns supported by immunohistochemical staining of human liver tissue. Differential gene expression patterns unique to PoPH nuclei, observed primarily in the region surrounding the central hepatic vein, also predicted enrichment of signaling pathways involved in GDF15 signaling and arginine metabolism. A distinct pattern of inflammatory signaling emerged when comparing differential gene expression between PoPH and non-PoPH cirrhosis liver tissue, and PoPH macrophage clusters exhibited a pro-inflammatory transcriptomic signature. We confirmed that circulating BMP9 levels discriminated PoPH from non-PoPH cirrhosis and IPAH. Taken together, this work reveals important new insights into how PoPH peri-central cellular dysfunction, diminished circulating BMP9, excess estrogen levels, altered arginine metabolism, inflammation, and dysregulated GDF15 signaling, might all potentially contribute to pulmonary vascular remodeling in PoPH.

The application of snRNAseq to human PoPH liver tissue identified multiple distinct clusters of hepatocytes, cholangiocytes, macrophages, LSEC’s, and HSC’s. Differential gene expression analysis showed relative increase in *ESR1* gene expression across multiple PoPH cell clusters, as well as decreased *BMPR2* expression in PoPH hepatocytes, relative to liver from non-PoPH patients with cirrhosis. Cell communication analyses identified augmentation of GDF pathway signaling, via release of GDF15, as unique to PoPH cells surrounding the liver central vein. Furthermore, pathway analysis suggested enrichment of estrogen and apelin signaling pathways in PoPH as compared to the non-PoPH cirrhosis liver, and biomarker analyses confirmed low levels of circulating BMP9 distinguished PoPH from non-PoPH cirrhosis and IPAH. These specific genes, proteins, and signaling pathways are notable given they have all been implicated previously in PAH and PoPH pathogenesis. Not only are *BMPR2* gene mutations the main genetic cause of heritable PAH, but diminished *BMPR2* gene expression is also widely observed in non-heritable subtypes of PAH^33^. Diminished BMPR2 signaling, combined with excess GDF ligands (including GDF8 and GDF11), is thought to promote pulmonary vascular remodeling in PAH by disrupting the normal balance of antiproliferative and pro-proliferative signaling that regulates the growth of pulmonary vascular smooth muscle and endothelial cells^34,35^. Increased *GDF15* expression has been shown in lung vascular endothelial cells from PAH relative to non-PAH, and increased circulating GDF15 has been shown to have prognostic value in pulmonary hypertension subtypes^36–38^. In non-hereditary PAH subjects, *BMPR2* gene expression has been shown to be lower in both peripheral blood mononuclear cells as well as lung vasculature as compared to healthy controls^39,40^. Evidence also suggests *BMPR2* expression is modulated by estrogen levels, as the *BMPR2* promoter binds to the estrogen receptor, and increased exogenous estrogen has been shown to reduce cell *BMPR2* expression in vitro^41^. Relevant to PoPH, clinical studies have shown increased circulating estrogen metabolites in PoPH relative to non-PoPH cirrhosis, and genetic variation in estrogen signaling genes has been associated with the risk of PoPH in patients with underlying liver disease^7,8^. Increased expression of the estrogen receptor has also been observed in the pulmonary arterioles of cirrhotic patients with histopathologic evidence of pulmonary vascular disease as compared to cirrhotic controls without pulmonary arterial vasculopathy, implying estrogen signaling may play a direct role in the process of pulmonary vascular remodeling in PoPH^25^. Estrogen signaling has been closely linked to apelin signaling, and in experimental PAH, estrogen-apelin interactions appear to exert a BMPR2-dependent cardioprotective effect on the right ventricle^18,42^. Studies have also identified BMP9, shown to circulate at diminished levels in PoPH plasma as compared to non-PoPH cirrhosis, as able to rescue BMPR2 deficiency in animal models^9,43^. These data, coupled with the findings from our study, suggests a plausible mechanism by which peri-central PoPH cellular dysfunction results in excess estrogen, GDF15 release, and a deficiency of BMP9 entering the central venous circulation. When combined with altered apelin signaling, these circulating intermediaries may disrupt the balance between BMPR2 and GDF ligands (such as GDF15) that control growth of pulmonary artery endothelial and smooth muscle cells, shifting towards a pro-proliferative state that stimulates pulmonary vascular remodeling and PoPH disease pathogenesis. Further confirmation of the importance of these proteins and pathways to PoPH disease pathogenesis is clearly warranted.

A distinct pattern of inflammatory signaling emerged when analyzing our snRNAseq differential gene expression data. Specifically, diminished expression of *IL32* and *IL34* was observed, along with increased expression of *IL4R* and *IL6R* across PoPH clusters. In addition, PoPH macrophage clusters displayed a unique inflammatory signature, characterized by increased expression of the pro-inflammatory *CXCL2* gene (a potent neutrophil chemoattractant), the activation marker *CD163*, and diminished *MIF* gene expression (which has been shown to have both pro- and anti-inflammatory effects on macrophages across different disease states)^44,45.46^. Taken together, this suggests PoPH liver tissue may be characterized by a distinct pattern of inflammatory signaling as compared to non-PoPH cirrhosis liver tissue, and macrophages may play an important role as effector cells in promoting a pro-inflammatory state. This finding is of particular interest given the strong association between pro-inflammatory cytokines (in particular IL6 and IL6R levels), pathobiology, and clinical outcomes in other forms of PAH^20,21,47^. However, the simultaneous finding of diminished expression of the pro-inflammatory *IL32* and *IL34* genes across multiple PoPH clusters, the lack of information regarding differential gene expression of other inflammatory immune cell populations (such as T, B, and NK cells), and the absence of pulmonary vascular tissue gene expression profiles in our study, all raise an important note of caution when interpreting these findings. Although our results provide a useful guide for understanding how key cell populations and signaling pathways might be involved in PoPH inflammation, additional transcriptomic and phenotypic comparison between lung and liver inflammatory cell populations in PoPH, and robust confirmation of the differential expression of key pro- and anti-inflammatory cytokines and chemokines in PoPH relative to non-PoPH cirrhosis, is needed to better clarify the role and importance of inflammation in PoPH pathogenesis.

The central hepatic vein, which drains directly into the vena cava, is anatomically the closest avenue of communication between the hepatic and pulmonary circulation. In addition, the liver lobule is known to be composed of distinct sub-populations of cells (endothelial, stellate, and hepatocytes) that demonstrate molecular and transcriptomic heterogeneity, consistent with spatially distinct cellular physiologic functions (liver “zones”) and responses to liver injury^28–32,48^. In our study, the differential expression pattern of *ESR1*, *BMPR2*, and *FLT1* in PoPH liver tissue as compared to non-PoPH liver tissue appeared to correlate with protein expression based on immunohistochemical staining of human liver tissue specimens. In particular, *BMPR2* expression appeared to be reduced, and both *ESR1* and *FLT1* expression appeared to be increased, in PoPH peri-central hepatocytes as compared to non-PoPH cirrhosis liver tissue. In addition, PoPH peri-central cells showed unique enhancement for GDF15 signaling and the arginine biosynthesis pathways, patterns absent when examining non-PoPH cells around the central vein and PoPH peri-portal cells. This latter finding is particularly notable because arginine is an amino acid precursor for the synthesis of the vasodilator nitric oxide (NO), which has long been implicated in the pathogenesis of pulmonary vascular disease^49,50^. Observations from experimental PAH animal models support a role for arginine in PAH, including that pulmonary artery endothelial cells from a PAH rodent model differentially expressed genes involved in the arginine biosynthesis pathway relative to cells from control rodents, and a deficiency in circulating arginine (with excess arginine intermediates) constitutes a plasma metabolite signature unique to the PAH monocrotaline rat model^51,52^. Human studies have corroborated this association, showing enrichment of arginine biosynthesis predicted by pathway analysis of intestinal microbiome gene expression in PAH subjects relative to non-PAH controls, and distinct levels of arginine metabolites and *ARG1* gene expression in human PAH whole lung tissue as compared to non-PAH lung tissue^53,54^. Although portal hypertension is required for PoPH disease pathogenesis, there is no known association between the degree of portal hypertension nor the extent of liver synthetic dysfunction and either the presence or severity of pulmonary arterial hypertension in PoPH; and the so-called “liver-lung” axis of molecular communication in PoPH remains incompletely defined^4,55,56^. Our work, in which transcriptomic, immunohistochemical, and biochemical pathway differences between PoPH and non-PoPH cirrhosis tissue were clustered around the central vein, suggests this region of the liver may play an outsized role in PoPH disease pathogenesis. Dysregulated peri-central hepatic arginine biosynthesis might result in a deficiency of pulmonary arterial NO production, augmenting and accelerating pulmonary vascular remodeling caused by disinhibited pulmonary arterial cells; arginine along with altered estrogen, GDF15, BMP9, and BMPR2 signaling may be key players in the “liver-lung” molecular crosstalk that drives PoPH pathogenesis. Further characterization of this region’s particular anatomic and functional characteristics will help clarify the true significance of the liver peri-central region to the development and progression PoPH, potentially identifying targetable mediators capable of disrupting this pathogenic process and ameliorating PoPH disease.

BMP9, a circulating vascular quiescence factor produced primarily by hepatic stellate cells, has been shown to be diminished in plasma from PoPH subjects as compared to those with non-PoPH cirrhosis^9–11,57^. Our work, in a larger cohort than previously studied, provides additional validation that circulating BMP9 levels may help discriminate PoPH from both non-PoPH cirrhosis and IPAH. Our results also support a potential mechanism by which low BMP9 could interact with GDF15 and the BMPR2 signaling pathway to promote vascular remodeling in PoPH. Using snRNAseq and pathway analysis, we failed to find strong evidence that low circulating BMP9 levels in PoPH were due to hepatic differential expression of the *GDF2* or *ENG* genes themselves. Further studies, examining the longitudinal changes in plasma BMP9 levels in PoPH both after targeted therapy and following liver transplantation, will be required to fully define both the significance and clinical utility of this biomarker in PoPH.

We did not observe a significant difference in circulating FLT1 levels between PoPH and non-PoPH cirrhosis groups in the second independent validation cohort, despite observing increased FLT1 immunohistochemistry staining in the PoPH liver peri-central region and elevated plasma FLT1 levels in PoPH subjects from the initial validation cohort. Given that a subset of plasma samples from the initial cohort were depleted and unable to be used for FLT1 measurement, and plasma samples from the initial validation cohort had been stored longer and had undergone more processing and freeze/thaw cycles than those from the second independent validation cohort, the differences in plasma FLT1 levels between the two cohorts may have been due to measurement error. In addition, as circulating FLT1 can be generated by either alternatively spliced mRNA that is directly translated into soluble FLT1 or post-translational cleavage of membrane-bound FLT1, and both the etiology and severity of liver disease may affect the levels of metalloproteinases and other FLT1 cleavage proteins, it is possible that the differences in plasma FLT1 levels between cohorts may be confounded by the differences in underlying etiology and severity of liver disease between PoPH and non-PoPH cirrhosis subjects across the two cohorts^58–60^.

Although our study is the first to describe the transcriptomic patterns of the human PoPH liver observed using single nuclear RNA sequencing, we acknowledge several limitations that are important to consider when interpreting the results of our work. Due to the rare nature of PoPH and the paucity of those affected to successfully undergo liver transplantation, we were only able to perform snRNAseq and IHC on a small number of human PoPH liver tissue samples. The resulting lack of diversity in race/ethnicity and sex in our cohort, and the heterogeneity in etiologies of underlying liver disease, limits the generalizability of our findings and may have affected our results. The limited sample availability of existing PAH biorepository resources precluded validation of all candidate biomarkers identified in the snRNAseq pathway analysis, and in isolation our transcriptomic data should be viewed as hypothesis-generating and in need of further validation. We measured plasma biomarkers in subjects with PoPH, IPAH, and non-PoPH cirrhosis, but did not include unaffected controls and cannot compare our circulating biomarker results to this group. We applied snRNAseq to our liver tissue samples to improve the yield of parenchymal (Hepatocyte) and mesenchymal cell (HSC’s and LSEC’s) transcripts that would have eluded capture with single-cell RNA sequencing, due to the difficulty in liberating cells embedded in the fibrotic extracellular matrix with single cell techniques. Because the single nuclear sequencing approach is known to partially obscure hepatic immune cell clusters (T-cells, B-cells, NK-cells), however, we may have missed important PoPH-specific differentially expressed genes, pathways, and biomarkers from these clusters^14^. Although our work provides valuable insights into the transcriptomic characteristics of the human PoPH liver, we do not examine gene expression patterns of the pulmonary vasculature unique to PoPH. True characterization of the “liver-lung” axis in PoPH would require an integrative approach examining lung and liver tissue in PoPH simultaneously, which is a vanishingly rare clinical opportunity.

In conclusion, data presented in this study provides evidence that the PoPH liver demonstrates unique patterns of gene expression as compared to the non-PoPH cirrhosis liver, and differential expression of the *BMPR2* and *ESR1* genes, enhanced signaling through the GDF15 signaling pathway, and enrichment of the arginine biosynthesis pathway are characteristics that are unique to PoPH cells surrounding the central hepatic vein. Taken together, our findings provide a framework for understanding how the liver peri-central region might influence liver-lung crosstalk and promote PoPH pathogenesis, and may ultimately lead to new targets and strategies for clinical trials.

## Methods

### Preparation of fresh tissue homogenates and nuclei from snap-frozen human liver tissue

Explanted human liver tissue was obtained at the time of liver transplantation. Samples were collected with institutional ethics approval from the University of Cincinnati IRB (UC IRB 2013–8157). All relevant ethical regulations were followed. PoPH was defined as precapillary PAH on resting right heart catheterization (mean pulmonary arterial pressure (mPAP) ≥25 mmHg, pulmonary capillary wedge pressure ≤ 15 mmHg, pulmonary vascular resistance (PVR) ≥3 Wood Units) that occurred in the context of portal hypertension (a measured hepatic venous pressure gradient ≥6 mmHg or sequelae of portal hypertension that included splenomegaly, thrombocytopenia, varices, or portal vein abnormalities) without other etiologies of PAH identified (such as underlying obstructive or interstitial lung disease, congenital heart disease, or chronic thromboembolic pulmonary vascular disease)^1–4^. Non-PoPH Cirrhosis was defined as patients with liver cirrhosis and sequelae of portal hypertension who had PAH ruled out by pre-operative echocardiography (estimated right ventricular systolic pressure < 30 mmHg) and confirmed by either pre-transplant or intra-operative RHC (mPAP < 20 mmHg, PVR < 3 Wood Units). After removal of the capsule, multiple samples of 5-mm cubed segments of tissue were obtained from different lobes of the explant and snap frozen in liquid nitrogen within 30 min of operative removal from the patient. Single nucleus suspensions were then generated using a modification of the “Humphrey’s” protocol for human kidney tissue (https://www.protocols.io/view/nuclei-isolation-from-human-kidney-for-single-nucl-81wgbobnlpko/v1)^61^. Nuclear lysis buffer 0 (NLB0) was created, composed of 10 ml of Nuclei EZ lysis buffer (Sigma Aldrich, St Louis, MO, USA) and 1 tablet of cOmplete ULTRA protease inhibitor (Sigma Aldrich, St Louis, MO, USA). Nuclear lysis buffer 1 (NLB1) was created, composed of 4 ml of NLB0, 20 μl of Promega RNAsin Plus (Fisher Scientific, Vilnius, Lithuania), and 20 μl of Invitrogen SUPERaseIN (Fisher Scientific, Vilnius, Lithuania), suspended in 0.1% Bovine Serum Albumin solution. Nuclei lysis buffer 2 was created, composed of 4 ml of Nuclei EZ lysis buffer (Sigma Aldrich, St Louis, MO, USA), 4 μl of Promega RNAsin Plus (Fisher Scientific, Vilnius, LT), and 4 μl of Invitrogen SUPERaseIN (Fisher Scientific, Vilnius, Lithuania), suspended in 0.1% Bovine Serum Albumin solution. Nuclei suspension buffer (NSB) was created, composed of 2 ml of Dulbecco’s phosphate buffered saline (Sigma Aldrich, St Louis, MO, USA) and 40 units/ml of Promega RNAsin Plus (Sigma Aldrich, St Louis, MO, USA), suspended in 1% Bovine Serum Albumin solution. Briefly, liver tissue cubes were placed on a 60 mm dish, 1 ml of NLB1 was added, and tissue was mechanically dissociated via mincing with a fresh razor blade. After transfer to a Dounce tissue grinder over ice, and addition of 1 ml of NLB1, further mechanical dissociation was performed via grinding 15–20 times with a loose pestle. The homogenate was passed through a 200 μl strainer and collected in a 50 ml conical tube. The homogenate was returned to a Dounce tissue grinder, and ground 7–10 times with a tight pestle. Following this, 2 ml of NLB1 was added, and the solution was incubated over ice for 5 min. The homogenate was passed through a 40 μm strainer into a 50 ml conical tube, then transferred to a 15 ml conical tube. This tube was centrifuged at 500 × *g* for 5 min at 4 °C. After discarding the supernatant, the resulting pellet was suspended in 4 ml of NLB2 and incubated on ice for 5 min. The tube was again centrifuged at 500 × *g* for 5 min at 4 °C. After again discarding the supernatant, the pellet was resuspended in 2 ml of NSB. The resulting suspension was passed through a 10 μm filter (pluriSelect Life Sciences, Leipzig, Germany) into a 50 ml conical tube.

### Sample processing, complementary DNA library preparation, sequencing, and data processing

Single nucleus samples were prepared per instructions provided in the 10X Genomics Single-Cell 3’v3.1 Reagent Kit. Following generation of single nucleus suspensions, examination was performed using trypan blue dye exclusion, to determine concentration and intact percentage of nuclei. For each sample, nuclei concentration was adjusted to 700–1200 nuclei/ul, 11200 nuclei were loaded into the 10X Chromium controller for 10X single nuclei sequencing (targeting recovery of 7000 nuclei), and a Gel Beads in Emulsion was generated. cDNA libraries were prepared as per instructions provided in the 10X Genomics Single-Cell 3’v3.1 Reagent kit, 18 cycles were used for cDNA amplification, and single nuclear libraries were sequenced using Illumina NovaSeq 6000. Single nuclear data were processed using the 10X Cell Ranger software version 6.1, mapping reads to a modified GRCh38 human genome which included intronic regions to ensure quantification of reads derived from immature messenger RNA (mRNA) present in the nucleus. Sequencing QC summaries for each liver profiled are found in Supplementary Table 5.

### Data integration, clustering, and differential gene expression analysis

Using the *Seurat* program version 4.0.6, the *scrublet* platform, and *R* version 4.1.2, data were processed. We excluded all genes detected in 10 or fewer nuclei, nuclei with fewer than 50 detected genes, nuclei with fewer than 1000 unique expressed genes (based on Barcode Rank Plots generated by the 10X CellRanger platform) and nuclei with greater than 10% of UMIs mapped to mitochondrial genome. Doublets were detected using the *scrublet* platform predicted doublet scores, with an expected doublet rate of 6%, and removed^62–64^. Each sample was size-factor normalized to 10,000 UMI per cell/nucleus, then log2 transformed and scaled using Seurat. Using data from all samples together, integration features were identified based on the top 2000 most highly variable features across all datasets. Ribosomal genes were removed from this list of highly variable features, and previously described canonical marker genes for human liver tissue single cell and single nuclear RNA sequencing (liver sinusoidal endothelial cells (*PECAM1, VWF, CD36, STAB1, STAB2, CALCRL*), hepatic stellate cells (*ACTA2, SPARC, DCN, COL1A2, COL4A2*), macrophages (*CD163, CD74, MARCO, MS4A7*), cholangiocytes (*SOX9, KRT7, ANXA4*), and hepatocytes (*ALB, TTR, CYP3A4, CYP3A7, PCK1, ARG1*) were added^13,28^. Each sample was then scaled and the top principal components were identified from individually scaled datasets. Finally, integration anchors across all datasets were identified using the *FindIntegrationAnchors* function in Seurat^63^, and samples were integrated via an anchor-based canonical correlation method. The integrated dataset was then scaled and the top 30 principal components were identified. An elbow plot was then constructed, which identified the top 15 principal components as optimal for clustering, and the integrated dataset was clustered using Seurat’s shared nearest neighbor (SNN)-Louvain clustering algorithm with 15 dimensions^65^. Using the Louvain modularity optimization in *Seurat*, the *clustree* package, and JackStraw plots, we explored clusters at several resolution values. With 15 principal components and a resolution of 1.8 we generated a total of 35 unique clusters for further analysis. Differentially expressed “marker” genes for a given cluster were identified using Seurat’s *FindAllMarkers* function, applying a likelihood ratio test and selecting upregulated genes expressed in a minimum of 25% of nuclei with a minimum log-fold difference in expression of 0.25 or greater between clusters. Clusters were named based on a combination of previously reported canonical marker genes, subjecting the top 500 differentially expressed genes to Gene Set Enrichment Analysis (GSEA), and manual curation^66^. GSEA analysis involved cross-referencing differentially expressed genes with known cell type signature gene sets, the degree of overlap based on false discovery adjusted hypergeometric testing^13,14,32,63,65^. Two clusters expressed a mix of marker genes, one identified with both hepatocyte and HSC markers, and another with a combination of cholangiocyte, HSC, and LSEC markers. These clusters of mixed marker genes were subsequently sub-clustered with 15 principal components at a resolution of 0.2, yielding sub-clusters which were subsequently named with the suffix “S1” based on the aforementioned marker genes. The final dataset consisted of 36 unique clusters, including multiple macrophage, hepatocyte, LSEC, and HSC cell clusters (Supplementary Data 1).

### Pathway analysis

Significant differentially expressed genes (identified by selecting a Benjamini-Hochberg corrected Wilcoxon Rank Sum test threshold of *p* < 0.05) were identified for each cluster by comparing PoPH and non-PoPH cirrhosis clusters using Seurat’s *findMarkers* function (Supplementary Data 1). The differential expression of genes previously associated with PoPH (*CYP19A1, CYP1B1, ESR1, BMP10, ENG, GDF2, BMPR2, ACVRL1*) was examined across various cell clusters^7–9^. Gene Ontology enrichment analysis was performed across clusters using Cytoscape version 3.9.1 and STRING enrichment^67^. Individual genes in a cluster were first scaled to the intensity of differential gene expression in PoPH relative to non-PoPH cirrhosis (log-fold-change between groups), STRING networks were constructed based on protein queries, functional enrichment was obtained for the largest discrete network, and significantly (false discovery rate corrected *p* < 0.05) affected KEGG pathways corresponding to this network were obtained. Canonical zonation markers were used to identify peri-central and peri-portal cells based on marker gene expression^12,29–31^. Predicted communication networks between cells and between groups were compared using the *CellChat* platform^68^. Briefly, *CellChat* produces predicted cell-cell communication networks by integrating gene expression data with known receptor-ligand interactions contained in the “CellChatDB” (a manually curated database of over 2000 receptor-ligand interactions in mouse and human), assigning significance of predicted enrichment based on the likelihood of differentially expressing a given receptor-ligand interaction pair when compared to a random permutation of receptor-ligand pairs. The resulting output assigns cell clusters as senders (ligands), receivers (receptors), or mediators/influencers (clusters that can control, potentiate, or suppress the communication between sender and receiver groups).

### Candidate circulating biomarker selection

The results of the KEGG pathway analysis was used to curate a genes that contributed to upregulated KEGG pathways of interest based on suspected mechanisms of PAH and PoPH vascular pathogenesis: estrogen signaling (hsa04915), TGF-B signaling (hsa04350), fluid shear stress and atherosclerosis (hsa05418), Hippo signaling (hsa04390), VEGF signaling (hsa04370), and HIF-1 signaling (hsa04066)^22–25^. Examining the differentially expressed genes corresponding to these predicted enriched pathways, and focusing on proteins known to be previously implicated as biomarkers in PAH, we selected the Vascular Endothelial Growth Factor Receptor 1 (FLT1) as a candidate biomarker for further validation. Biomarkers known to be associated with PAH (serum NTproBNP) and PoPH (plasma BMP9) were also selected as reference comparators to help quantify the potential diagnostic and/or prognostic utility of novel candidate PoPH-specific biomarkers^9,10^.

### Biomarker analysis validation cohort

The National Biological Sample and Data Repository for PAH (“PAH Biobank”) is a National Institutes of Health-funded repository of biological samples and clinical data collected from 37 enrolling PAH centers across the United States. Patients with World Health Organization Group 1 PAH, based on right heart catheterization (RHC) hemodynamics and reviewed by pulmonary hypertension specialists, were screened for enrollment. Biorepository data collection was approved by the institutional review board at each participating center, and all patients gave informed consent at the time of enrollment^69^. All relevant ethical regulations were followed. PAH was defined hemodynamically as a mPAP > 25 mmHg, pulmonary capillary wedge pressure or left ventricular end-diastolic pressure ≤15 mmHg, and PVR ≥ 3 Wood units (240 dyne•s•cm^−5^) on resting RHC. As part of the original repository protocol, site principal investigators classified PoPH as PAH occurring in the context of underlying portal hypertensive liver disease, and Idiopathic PAH (IPAH) as PAH occurring with no identifiable cause. Portal hypertensive liver disease was determined either clinically based on the presence of classic sequelae (varices, portal hypertensive gastropathy, hepatorenal syndrome, spontaneous bacterial peritonitis) or by direct measurement of an elevated hepatic venous pressure gradient. Demographic information (age, sex, race/ethnicity) and body mass index (BMI, kg/m^2^) were collected upon enrollment. Non-fasting blood samples were collected at the time of study enrollment via peripheral venous phlebotomy using serum separator tubes or sodium heparin plasma tubes. Following centrifugation, aliquots of serum and plasma were stored at −80 °C. From the PAH biobank, we randomly selected 80 out of the total of 192 PoPH subjects with a full complement of biological samples and clinical data. We also selected 40 IPAH subjects as controls, to be included in the initial validation cohort.

Our institution routinely collects biological samples and clinical data from adult patients with known or suspected liver disease (“UC Cirrhosis Biobank”) (Approved by the University of Cincinnati IRB, UC IRB 2012-3689). All participants provide informed consent at the time of enrollment. All relevant ethical regulations were followed. Occult pulmonary vascular disease was assessed by transthoracic echocardiography, and non-PoPH cirrhosis defined by liver cirrhosis in the absence of echocardiographic pulmonary hypertension (tricuspid regurgitant jet velocity below 2.8 m/s, tricuspid annular plane systolic excursion (TAPSE) value greater than 2 cm, and ratio of right-to-left ventricular diastolic diameter below 0.8)^1,2^. Non-fasting blood samples were collected at the time of study enrollment via peripheral venous phlebotomy, using serum separator tubes or sodium heparin plasma tubes. Following centrifugation, aliquots of serum and plasma were stored at −80 °C. We selected 50 non-PoPH Cirrhosis subjects from the UC Cirrhosis Biobank matched on age and sex to PoPH subjects, and obtained blood samples (serum and plasma) and clinical data from these subjects, to be included in the initial validation cohort.

The Pulmonary Vascular Complications of Liver Disease 2 (PVCLD2) study enrolled patients evaluated for liver transplantation or pulmonary hypertension across 8 centers in the United States between 2013 and 2017 (“PVCLD2 Biobank”). Study participation was approved by the institutional review board at each participating center, and all patients gave informed consent at the time of enrollment. All relevant ethical regulations were followed. The only inclusion criterion was the presence of chronic portal hypertension with or without intrinsic liver disease. Patients with evidence of active infection, gastrointestinal bleeding in the two weeks prior to study enrollment, or prior liver or lung transplantation were excluded. The diagnosis of PoPH in liver cirrhosis was based on a combination of transthoracic echocardiography, pulmonary function testing, imaging, and clinical assessment by PAH specialists. Specifically, PoPH was defined by RHC hemodynamics (mPAP > 25 mmHg, pulmonary capillary wedge pressure or left ventricular end-diastolic pressure ≤15 mmHg, and PVR ≥ 3 Wood units (240 dyne•sec•cm^−5^)) without another etiology of pulmonary hypertension present. Patients with a significant obstructive or restrictive ventilatory defect on pulmonary function testing performed according to American Thoracic Society and European Respiratory Society recommendations (forced expiratory volume in one second (FEV_1_) divided by forced vital capacity (FVC) below 70%, with FEV1 of less than 50% predicted or FVC less than 60% predicted, using standard sex-specific, age-specific, and race-specific reference equations) were excluded^1,2,8^. The Model for End-stage Liver Disease (MELD) score was calculated for patients using the formulas: MELD = 10*{[0.957*ln(creatinine] + [0.378*ln(bilirubin)] + [1.12*ln(international normalized ratio]} + 6.43; and MELD-Na = MELD + [0.32*(137-sodium)] − [0.033*MELD*(137-sodium)]^70^. Patients with HIV infection and those with significant valvular heart disease (mitral or aortic) or left ventricular diastolic dysfunction on transthoracic echocardiography were excluded. Non-PoPH cirrhosis patients were those with known portal hypertension (determined clinically or directly measured via the hepatic venous pressure gradient) with transthoracic echocardiographic testing showing a right ventricular systolic pressure less than 40 mmHg and the absence of right ventricular dysfunction. A single standardized echocardiographic protocol was applied to all subjects, and the Echocardiography Core Laboratory at the Mayo Clinic evaluated all individual echocardiograms. The institutional review boards at each of the participating centers approved biorepository data collection, and informed consent was obtained from all patients at the time of enrollment. Blood samples were collected at time of study enrollment via peripheral venous phlebotomy after an overnight fast, using serum separator tubes or sodium heparin plasma tubes. Following centrifugation, aliquots of serum and plasma were stored at −80 °C. Out of the full cohort of 454 subjects enrolled in the PVCLD2 biobank, a total of 327 subjects had available data and were included in the final biobank cohort, of which 37 had PoPH and 290 were non-PoPH cirrhosis controls. We created a second independent validation cohort comprised of 25 PoPH and 75 non-PoPH cirrhosis subjects randomly selected from the PVCLD2 Biobank.

### Biomarker measurement

Plasma FLT1 was measured using a commercially available multiplex magnetic bead immunoassay kit (Milliplex Immunoassay HANG2MAG-12K-01, S MilliporeSigma, Burlington MA). Plasma BMP9 was measured using a magnetic bead immunoassay kit (Luminex Immunoassay LXSAHM-03, R&D Systems, Minneapolis, MN) in the initial cohort. NTproBNP serum levels were measured using a commercially available multiplex magnetic bead immunoassay kit (Milliplex Immunoassay HCVD1MAG-67K-01, MilliporeSigma, Burlington, MA).

### Immunohistochemistry

Unstained formalin-fixed paraffin-embedded (FFPE) liver tissue sections (2 μm thickness) were obtained from archival tissue obtained at the time of surgical resection or transplantation. Liver tissue was obtained from three PoPH and five non-PoPH cirrhotic patients, and three non-cirrhotic control patients who had liver tissue removed for bleeding, trauma, and a ruptured adenoma. The same hemodynamic and clinical definitions used to identify PoPH and non-PoPH cirrhosis subjects for snRNAseq analysis of liver tissue were used to characterize subjects for archival liver tissue immunohistochemistry. Non-cirrhosis controls were defined by the absence of known pulmonary vascular or liver disease at the time of surgery. Immunohistochemical study of the expression levels of molecular markers FLT1 (VEGFR1), BMPR2, and the estrogen receptor ESR1 was carried out using the following primary antibodies: (i) recombinant rabbit anti-VEGF Receptor 1 monoclonal IgG antibody (ab32152, Abcam, Great Britain), (ii) recombinant rabbit anti-Estrogen Receptor alpha monoclonal IgG antibody (ab108398, Abcam, Great Britain), (iii) recombinant rabbit anti-BMPR2 polyclonal IgG antibody (ab124463, Abcam, Great Britain); and secondary antibody: (iv) unconjugated polyclonal rabbit anti-goat IgG (H + L) secondary antibody (PISA510312, Fisher Scientific, Vilnius, Lithuania). Tissue samples were first deparaffinized by washing in distilled water, followed by antigen retrieval using a citrate buffer solution. Sections were then blocked first with BLOXALL endogenous peroxide blocker (Vector Laboratories BLOXALL endogenous peroxide and alkaline phosphatase blocking solution 100 ml, NC0185217, Fisher Scientific, Vilnius, Lithuania). Primary staining (FLT1 antibody at a dilution of 1:200, BMPR2 antibody at a dilution of 1:75, and ESR1 antibody at a dilution of 1:250) was applied overnight, followed by secondary staining using the rabbit anti-goat antibody listed above at a dilution of 1:500. Slides were then developed using DAPI (Vector Laboratories DAPI, Fisher Scientific, Vilnius, Lithuania), countered with hematoxylin, and dehydrated.

The qualitative assessment of the expression of molecular markers was carried out by analyzing digital images obtained with an Olympus BX60 optical microscope using the pre-installed *cellSens* software and an Olympus DP72 digital camera (Olympus Corporation, Tokyo, Japan). Images of both the portal triad region and the central vein region were obtained at 20x magnification for each subject. Limited tissue samples prevented us from testing BMPR2 and ESR1 in NL3 samples. Semi-quantitative immunohistochemistry was performed using the Fiji software analysis platform ImageJ2 version 2.9.0/1.53t^71,72^ (Supplementary Data 1).

Briefly, digital images were first subject to color deconvolution using the ImageJ2 platform. The antibody staining image threshold was then adjusted to select only the antibody signal, without capturing the background signal, and measured. The hematoxylin-stained image threshold was also adjusted, and the number of nuclei in the image counted using the automated software. Finally, a value of the semi-quantitative staining was generated by normalizing the mean antibody staining intensity for the number of nuclei counted.

### Statistics and reproducibility

Continuous variables are presented as median with interquartile ranges (IQR), categorical variables summarized using number and percentage (%). Mann–Whitney–Wilcoxon tests and Chi-Squared tests were used as appropriate.

Missing biomarker levels were removed in a pairwise fashion prior to analysis. All biomarker levels were measured in duplicate (Supplementary Data 1). The lower limit of detection for plasma BMP9 was 2.06 pg/ml. Serum NTproBNP levels, below the limit of detection in four non-PoPH cirrhosis subjects from the initial validation cohort, were imputed as 1/10th of the lower limit of detection. Owing to sample depletion, some subjects were missing either serum or plasma samples for biomarker testing (Supplementary Table 6). For the initial validation cohort of 80 PoPH and 50 non-PoPH Cirrhosis subjects, intra-assay coefficient of variation for NTproBNP was 8.7%, and for plasma FLT1 and BMP9 were 7.7% and 9.8%, respectively. For the second independent validation cohort of 25 PoPH, 75 non-PoPH Cirrhosis subjects from the PVCLD2 biorepository, inter-assay coefficient of variation for serum NTproBNP was 17.4%. Plasma FLT1 testing on the PVCLD2 cohort was conducted off-site, and inter-assay coefficients of variation were not readily available. For the final cohort of 40 IPAH subjects, intra-assay coefficient of variation for plasma BMP9 was 6.6%.

As the assumption of normality was violated for distributions of biomarker levels and hemodynamic parameters, relationships between biomarker levels and PoPH disease severity (mPAP, Cardiac Index (CI), and PVR obtained on right heart catheterization) were assessed using the non-parametric Kendall Correlation. Analyses of biomarker relationships with disease etiology (PoPH or non-PoPH cirrhosis) were performed using logistic regression models. Biomarker levels were log-transformed prior to logistic regression analysis. Univariate regression models were adjusted in a multivariable fashion for clinical covariates (age, sex, race/ethnicity, and body mass index (BMI)) in the initial validation cohort comprised of PoPH, non-PoPH cirrhosis, and IPAH subjects.

Semi-quantitative immunohistochemistry staining values were compared between tissue types (non-cirrhosis control (*N* = 3), PoPH (*N* = 3), non-PoPH cirrhosis (*N* = 5) for the peri-portal region and peri-central region of liver tissue, without statistical testing performed.

All analyses were performed using R (version 4.1.2, R. Foundation for statistical computing, Vienna, Austria).

### Reporting summary

Further information on research design is available in the Nature Portfolio Reporting Summary linked to this article.

### Supplementary information

Supplemental Material

Description of Additional Supplementary Files

Supplemental Data 1

Reporting Summary

## Data Availability

All single nucleus RNA sequencing data generated by this study have been deposited in the Mendeley Data archive (Jose, Arun (2023), “Human single nuclear RNA seq PoPH and non-PoPH Cirrhosis Liver”, Mendeley Data, V1, doi: 10.17632/y2s67z8tw9.1). The data supporting the findings of this study are available within the paper and its Supplementary Information.
