# Supplementary material for: Human liver single nuclear RNA sequencing implicates BMPR2, GDF15, arginine, and estrogen in portopulmonary hypertension
Source: Commun Biol. 2023 Aug 9;6:826. doi: 10.1038/s42003-023-05193-3 (PMC10412637; doi:10.1038/s42003-023-05193-3)
Supplement: Supplementary file 1 — Supplemental Material [file 42003_2023_5193_MOESM1_ESM.pdf]

Supplementary Information for

Single Nuclear RNA Sequencing of Liver from Patients with Portopulmonary Hypertension

Arun Jose MD MS, Jean M. Elwing MD, Steven M. Kawut MD MS, Michael W. Pauciulo MBA, Kenneth E. Sherman MD PhD, William C. Nichols PhD, Michael B. Fallon MD, Francis X. McCormack MD, and the Pulmonary Vascular Complications of Liver Disease (PVCLD2) Study Group

This file includes:

Supplementary Figures 1-11

Supplementary Tables 1-6

List of PVCLD2 Study Group consortium members

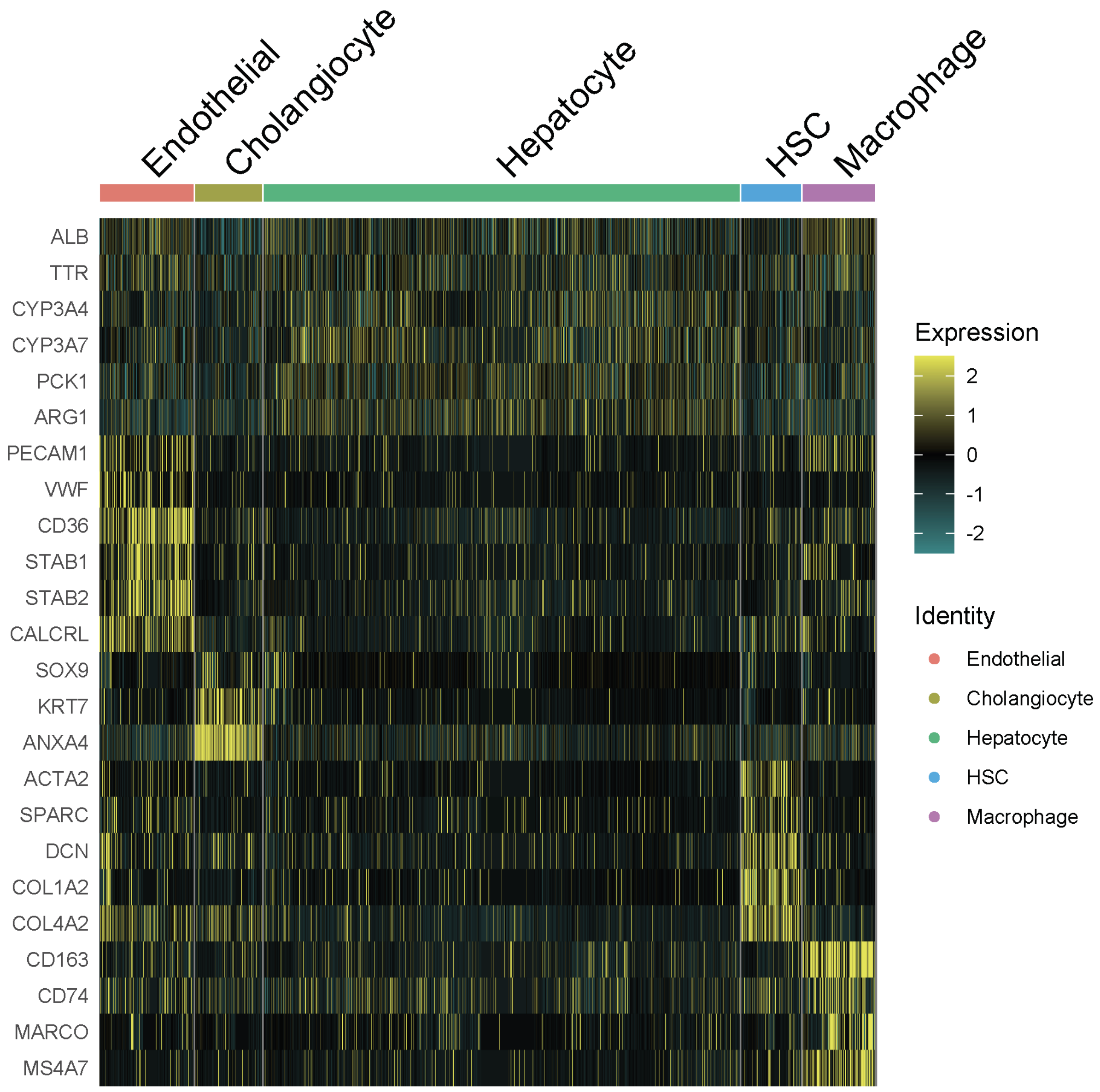

Supplementary Figure 1: Heatmap of Marker Genes and Clusters for snRNAseq

Heatmap showing cluster marker genes (Y-axis) and cluster identities (X-axis, top) for all named clusters identified by snRNAseq analysis. Normalized gene expression strength is color-coded. Major clusters of endothelial, cholangiocyte, HSC, and macrophages are clearly visible by high differential gene expression of marker genes.

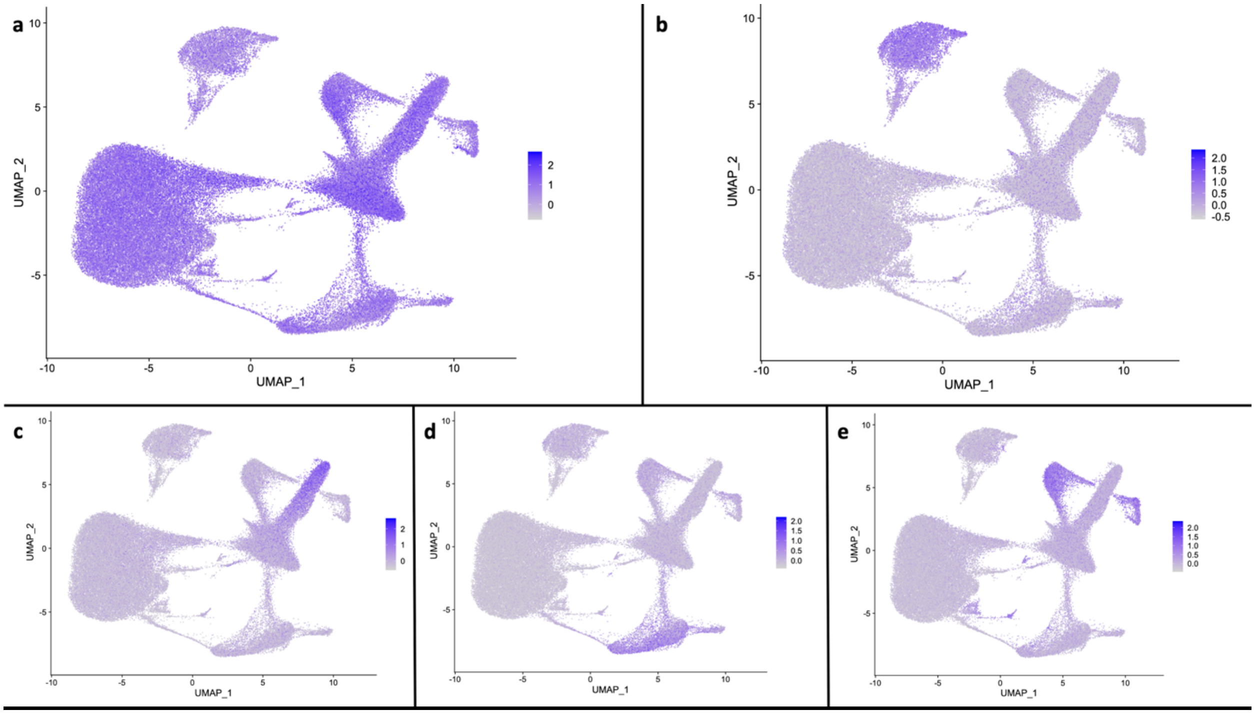

Supplementary Figure 2: Differential Expression of Cluster Marker Genes

UMAP projection of dataset highlighting canonical marker genes for major liver cell types including hepatocytes (*ALB, TTR, CYP3A4, CYP3A7, PCK1, ARG1,* panel a), cholangiocytes (*SOX9, KRT7, ANXA4*, panel b), macrophages (*CD163, CD74, MARCO, MS4A7,* panel c), HSC (*ACTA2, SPARC, DCN, COL1A2, COL4A2*, panel d), and endothelial cells (PECAM1, VWF, CD36, STAB1, STAB2, CALCRL, panel e).

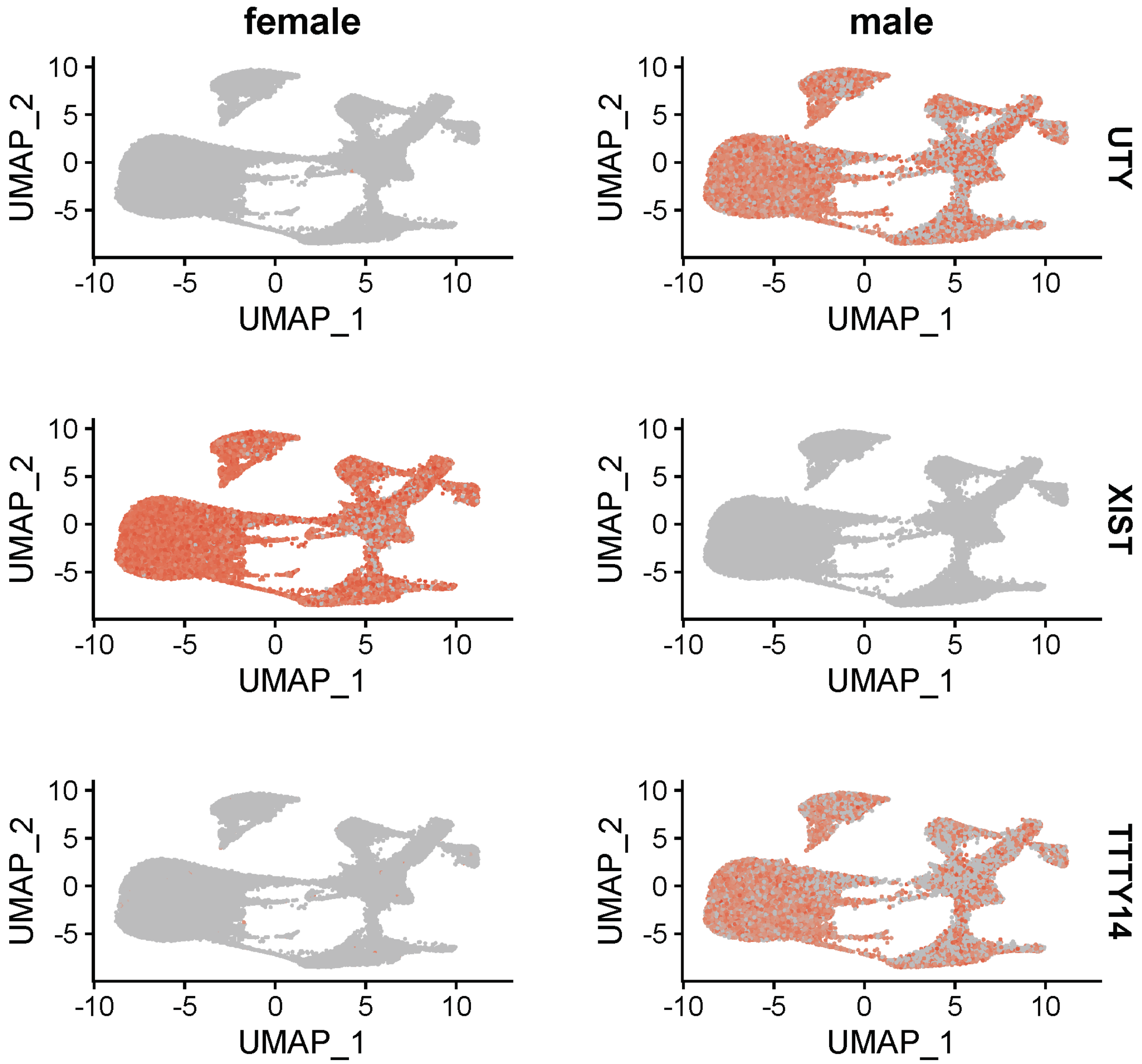

Supplementary Figure 3: Differential Expression of Sex-specific Genes

UMAP projection of nuclei derived from PoPH and non-PoPH cirrhosis tissue, separated into female (left) and male (right) columns. Increased expression of sex-specific genes *UTY, XIST*, and *TTTY14* displayed in red.

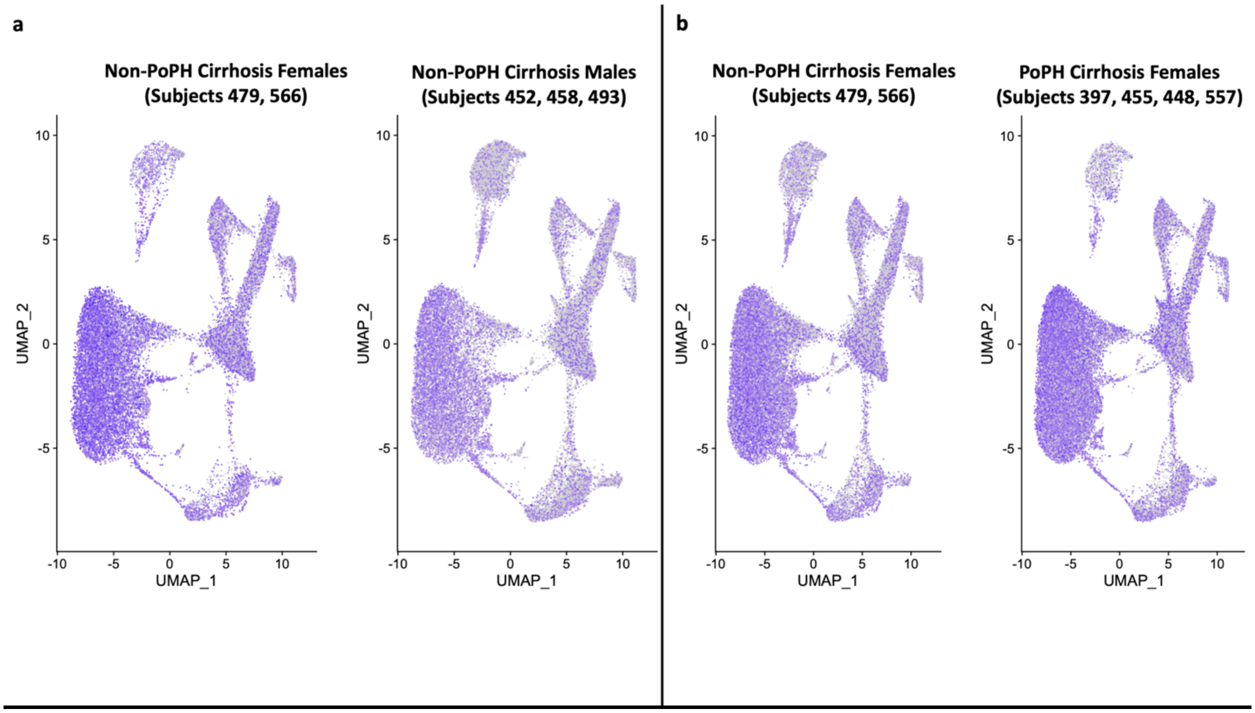

Supplementary Figure 4: Differential Expression of *ESR1* Across Different Groups

UMAP projection of *ESR1* gene expression in non-PoPH cirrhosis subjects grouped by sex (female: subjects 479 and 566; male: subjects 452, 458, and 593) shows increased expression in female subject hepatocyte clusters (panel a). *ESR1* gene expression was also compared between female subjects with PoPH (subjects 397, 455, 448, and 557) and female subjects with non-PoPH cirrhosis (subjects 479 and 566), showing increased differential expression in PoPH subject hepatocyte clusters (panel b).

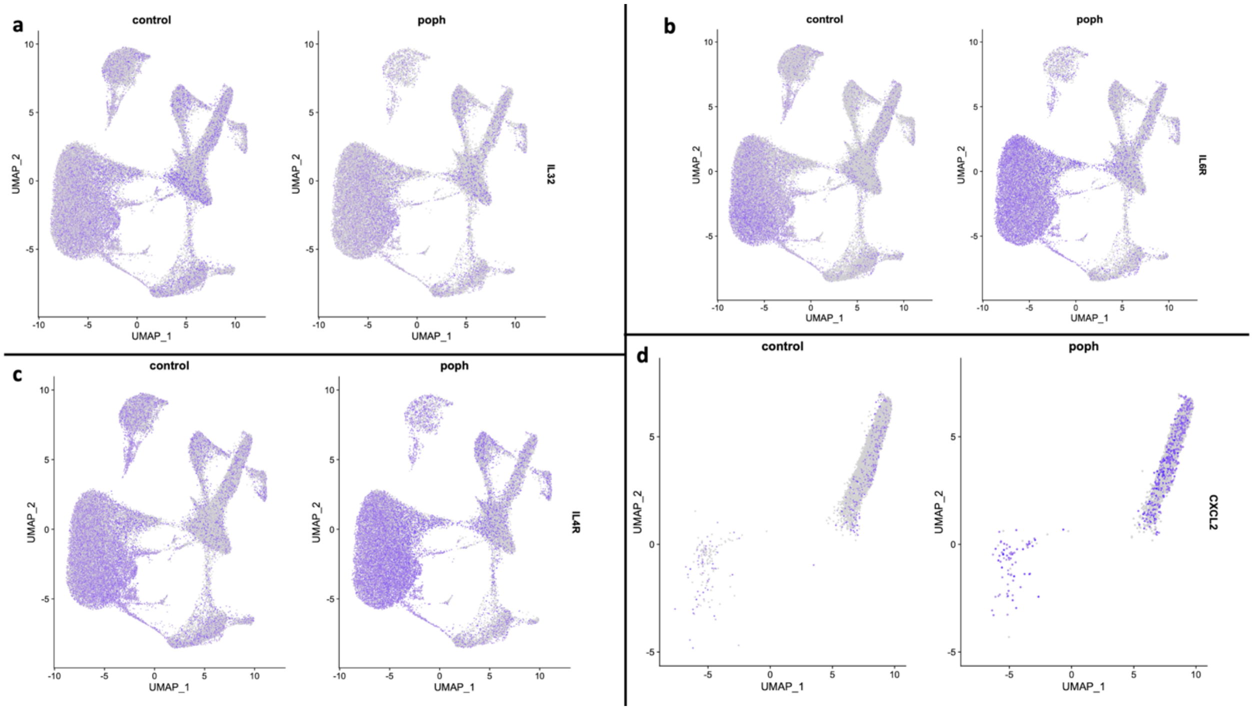

Supplementary Figure 5: Differential Expression of Select Inflammatory Cytokine and Chemokine Genes

UMAP projection demonstrating decreased expression of *IL32* gene in PoPH clusters relative to non-PoPH cirrhosis (panel a). Conversely, there is increased expression of the *IL6R* (panel b) and *IL4R* (panel c) genes in PoPH clusters relative to non-PoPH cirrhosis. PoPH macrophage clusters demonstrate strong differential expression for the pro-inflammatory neutrophil attractant gene *CXCL2* (panel d).

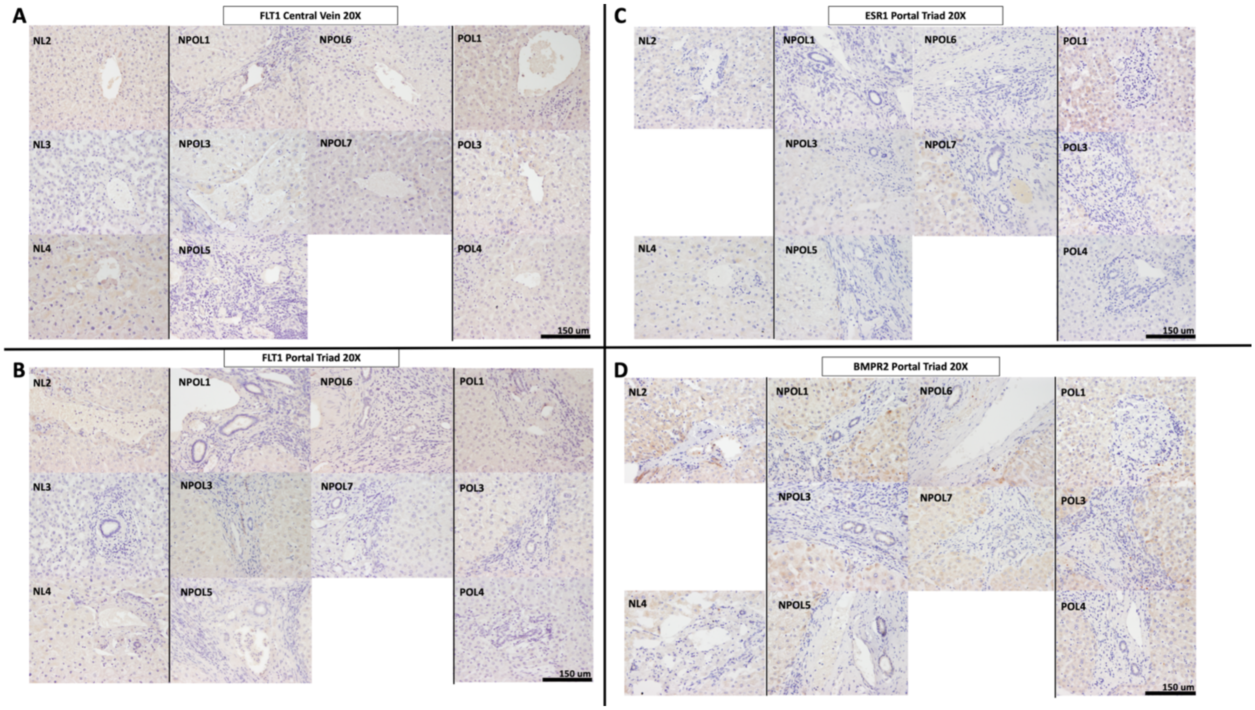

Supplementary Figure 6: Immunohistochemistry of Human Liver Tissue Samples for FLT1 and Portal Triad Region

No significant difference in FLT1 expression is seen between PoPH and non-PoPH cirrhosis samples in either the region surrounding the central vein (Panel a) or the region around the portal triad (Panel b). No differences in either ESR1 (Panel c) or BMPR2 (Panel d) expression are seen between PoPH and non-PoPH cirrhosis samples in the region around the portal triad. Note that limited tissue samples prevented us from testing BMPR2 in NL3 samples.

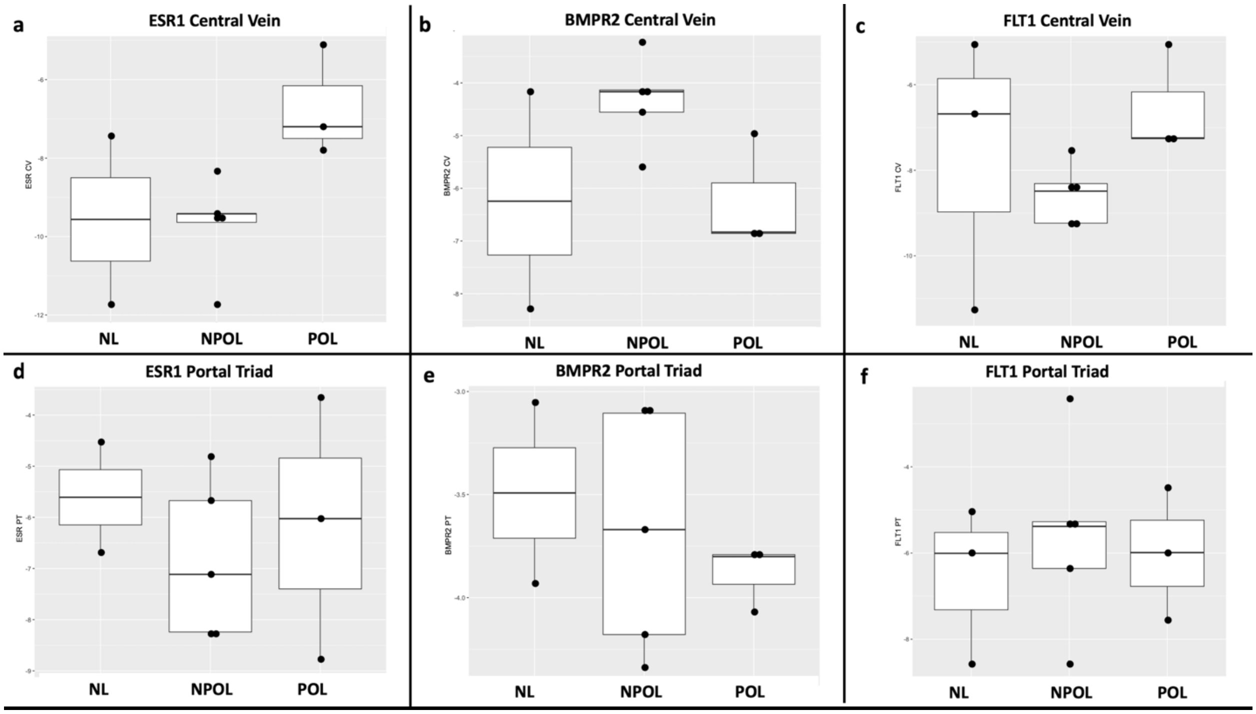

Supplementary Figure 7: Semi-Quantitative Immunohistochemistry Results

Dot plots showing increased semi-quantitative immunohistochemistry staining for ESR1 (Panel a) and FLT1 (Panel c), and decreased staining for BMPR2 (Panel b) in PoPH tissue (POL) as compared to non-PoPH cirrhosis liver tissue (NPOL) in the peri-central region. No difference is seen in ESR1 (Panel d), BMPR2 (Panel e), or FLT1 (panel f) staining in the peri-portal region between PoPH and non-PoPH liver tissue. Non-cirrhotic healthy liver tissue (NL) is also depicted for reference. Note that semi-quantitative immunohistochemistry results are displayed on a log-transformed Y-axis, with median (bold horizontal line) and interquartile range (box) demonstrated.

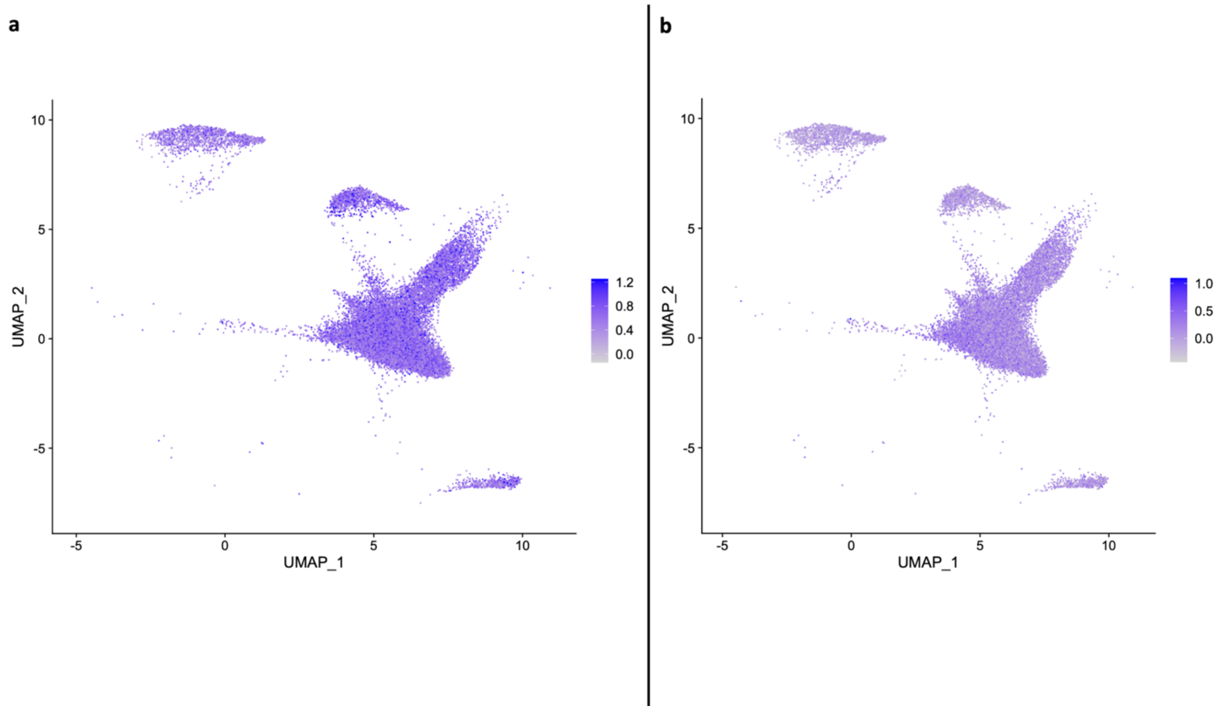

Supplementary Figure 8: Differential Expression of Zone-specific Genes for "Peri-Portal Zone" Clusters

UMAP projection showing zone-specific genes for "peri-portal zone" clusters of cells (Hepatocyte 1 and 2, HSC 4, LSEC 3, Cholangiocyte 3, and Macrophage 1 clusters). Increased expression of portal zone specific genes (*ALB, EFNB2, MSR1, NTN4, JAG1, EPHX1, EPCAM*, panel a) and decreased expression of central vein specific genes (*RSPO3, LGR5, FABP4, NOTUM, TBX3, GLUL, G6PC, GHR, CYP2E1, ZNRF3*, panel b) is observed.

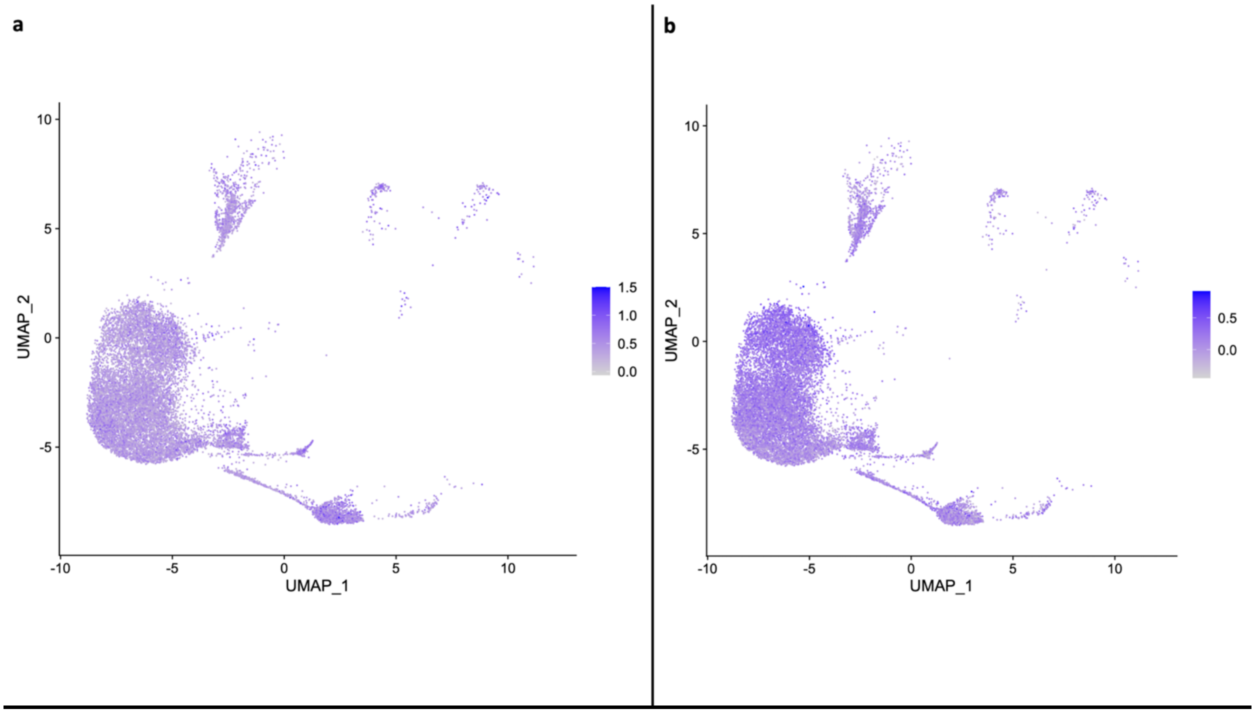

Supplementary Figure 9: Differential Expression of Zone-specific Genes for "Peri-Central Zone" Clusters

UMAP projection showing zone-specific genes for "peri-central zone" clusters of cells (Hepatocyte 5, Hepatocyte 9, Hepatocyte 10, HSC 2, HSC S1, LSEC 4, LSEC 6, Macrophage 4, and Cholangiocyte 4 clusters). Decreased expression of portal zone specific genes (*ALB, EFNB2, MSR1, NTN4, JAG1, EPHX1, EPCAM*, panel a) and increased expression of central vein specific genes (*RSPO3, LGR5, FABP4, NOTUM, TBX3, GLUL, G6PC, GHR, CYP2E1, ZNRF3*, panel b) is observed.

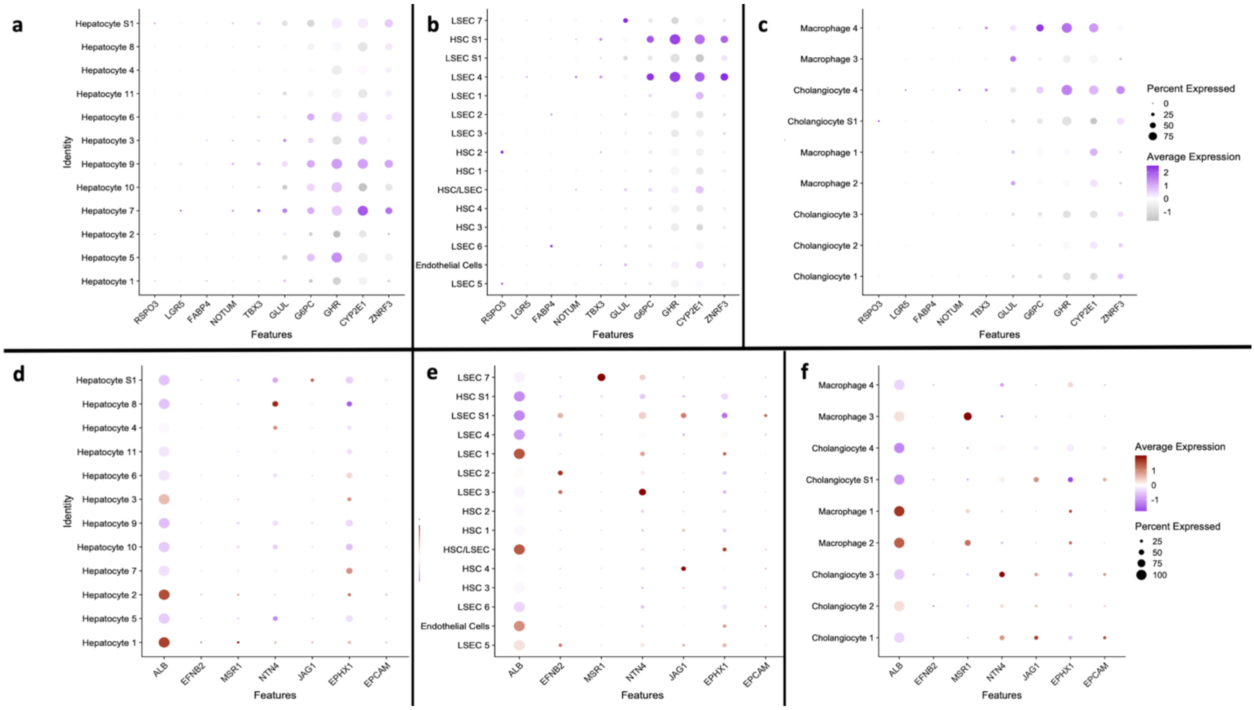

Supplementary Figure 10: Differential Expression of Zone-specific Genes in PoPH

Dot Plot showing differential gene expression of zone-specific genes in PoPH samples, color-coded by average gene expression, with the size of the dots reflective of the percentage of cells within a given cluster expressing a given gene. Genes associated with a peri-central location are upregulated (purple shading) in select clusters of hepatocytes (Panel a), endothelial and stellate cells (Panel b), and macrophage and cholangiocytes (Panel c). The upregulated expression of genes associated with a peri-portal location (red shading) is demonstrated for hepatocytes (Panel d), endothelial and stellate cells (Panel e), and macrophage and cholangiocytes (Panel f). Clusters believed to be peri-central were identified based on increased peri-central gene expression with diminished or absent peri-portal gene expression (Hepatocyte 5, 9, and 10; HSC 2 and S1; LSEC 4 and 6; Macrophage 4; Cholangiocyte 4), and peri-portal clusters by the opposite pattern (Hepatocyte 1 and 2; HSC 4; LSEC 3; Cholangiocyte 3; Macrophage 1).

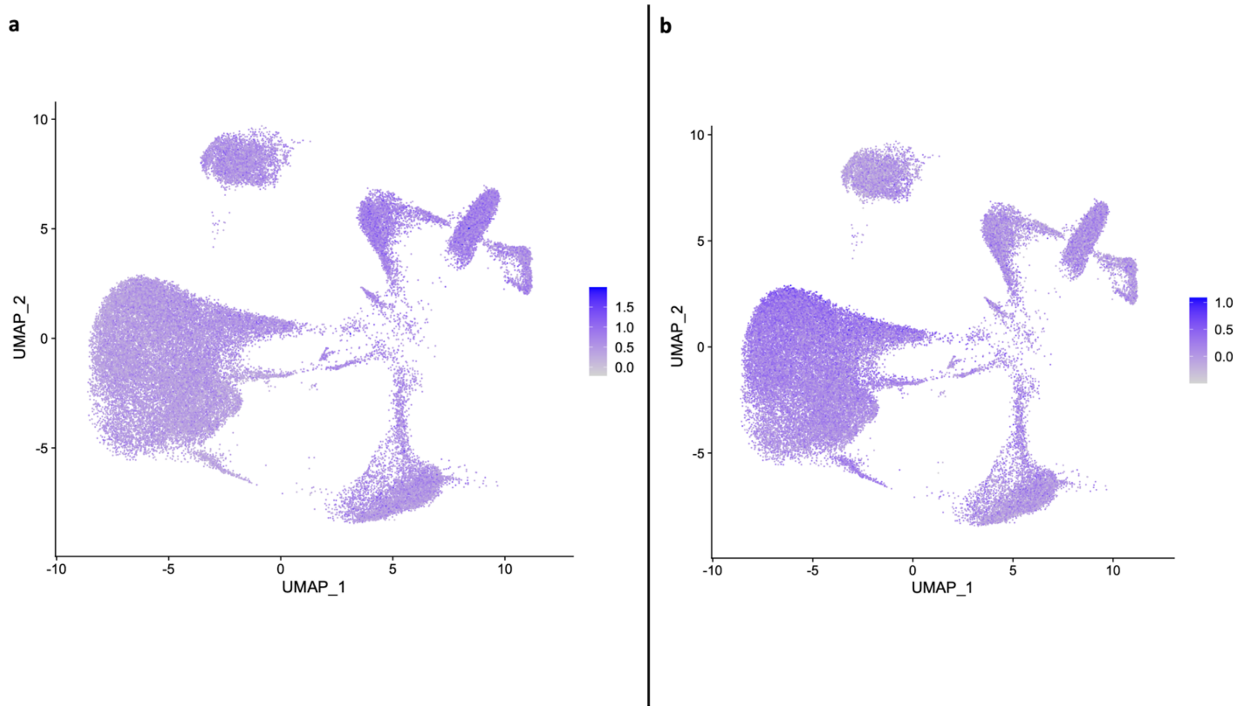

Supplementary Figure 11: Differential Expression of Zone-specific Genes for "Intermediate Zone" Clusters in PoPH

UMAP projection showing zone-specific genes for "intermediate zone" clusters of cells (Hepatocyte 3, Hepatocyte 4, Hepatocyte 6, Hepatocyte 7, Hepatocyte 8, Hepatocyte 11, Hepatocyte S1, HSC 1, HSC 3, LSEC 1, LSEC 2, LSEC 5, LSEC 7, LSEC S1, HSC/LSEC, Endothelial Cells, Cholangiocyte 1, Cholangiocyte 2, Cholangiocyte S1, Macrophage 2, and Macrophage 3 clusters). No obvious difference between expression of portal zone specific genes (*ALB, EFNB2, MSR1, NTN4, JAG1, EPHX1, EPCAM*, panel a) and expression of central vein specific genes (*RSPO3, LGR5, FABP4, NOTUM, TBX3, GLUL, G6PC, GHR, CYP2E1, ZNRF3*, panel b) is observed.

Supplementary Table 1: Differential Expression of Select Genes (BMPR2, ESR1, and FLT1) and Pathway Analysis Across Clusters

Differential Gene Expression Across Clusters			
Gene	Average Log-2 Fold Change Between Groups	FDR-Adjusted P-value	Cluster
BMPR2	-0.334	3.3E-30	Hepatocyte 5
	-0.268	9.8E-12	Hepatocyte 6
	-0.316	1.5E-19	Hepatocyte 8
	-0.293	1.1E-13	Hepatocyte 9
	-0.350	1.1E-13	Hepatocyte 10
ESR1	0.318	2.3E-02	Macrophage 1
	0.692	1.4E-04	Cholangiocyte 2
	0.693	3.6E-99	Hepatocyte 1
	0.411	5.4E-25	Hepatocyte 3
FLT1	0.514	4.5E-04	HSC 1
	0.479	2.0E-03	HSC 3
	0.830	8.5E-04	HSC 4
	0.659	9.8E-17	Endothelial
	0.370	1.0E-02	Cholangiocyte 1
	0.893	4.1E-16	Cholangiocyte 2
	0.962	5.7E-132	Hepatocyte 1
	0.358	2.4E-03	Hepatocyte 2
GDF15	0.044	2.2E-10	Hepatocyte 9
	0.442	5.1E-05	Hepatocyte S1
ESR1 (female only)	0.289	3.7E-11	Hepatocyte 1
	0.354	1.3E-16	Hepatocyte 3
KEGG Pathway Enrichment Across Clusters			
KEGG	Description	FDR adjusted p-value	Clusters
hsa04915	Estrogen Signaling Pathway	4.6E-02	Endothelial
		1.0E-02	Hepatocyte 1
		3.7E-02	Hepatocyte 4
		4.3E-02	Hepatocyte S1
		2.4E-03	LSEC 1
		9.4E-03	LSEC 2
		3.6E-03	Macrophage 1
hsa05418	Fluid Shear Stress & Atherosclerosis	3.1E-02	Cholangiocyte 2
		3.2E-03	Macrophage 1
		3.2E-03	Macrophage 2
		1.8E-02	Macrophage 4
		4.2E-03	Endothelial
		7.9E-06	Hepatocyte 1
		2.0E-02	Hepatocyte 2
		4.8E-03	Hepatocyte 3

		7.0E-04	Hepatocyte 4
		3.8E-03	Hepatocyte 5
		1.4E-03	Hepatocyte 6
		4.0E-04	Hepatocyte 7
		6.8E-06	Hepatocyte 8
		2.1E-03	Hepatocyte 9
		5.1E-03	Hepatocyte 10
		3.8E-03	Hepatocyte 11
		2.4E-02	Hepatocyte S1
		4.4E-02	HSC 1
		2.7E-02	HSC 2
		2.2E-02	HSC 3
		3.0E-02	HSC/LSEC
		2.0E-05	LSEC 1
		2.4E-04	LSEC 2
		2.8E-03	LSEC 3
		1.1E-02	LSEC 4
hsa04390	Hippo Signaling Pathway	2.0E-02	Cholangiocyte 4
		4.6E-02	Hepatocyte 1
		4.7E-02	Hepatocyte 8
		1.6E-02	Hepatocyte 9
		2.4E-02	Hepatocyte S1
hsa04350	TGF-beta Signaling Pathway	2.4E-02	Hepatocyte S1
		5.0E-02	HSC/LSEC
hsa04371	Apelin Signaling Pathway	3.8E-02	Cholangiocyte 1
		9.2E-04	Cholangiocyte 2
		1.0E-02	Endothelial
		3.1E-02	Hepatocyte 1
		2.5E-02	Hepatocyte S1
		4.0E-03	HSC 1
		1.5E-03	HSC 2
		1.4E-02	HSC 3
		5.0E-02	HSC/LSEC
		2.2E-02	LSEC 1
		3.3E-02	LSEC 2
		4.3E-02	LSEC 3
		3.5E-02	LSEC 5
		3.7E-02	Macrophage 1
		1.1E-02	Macrophage 2
hsa04370	VEGF Signaling Pathway	4.0E-02	LSEC 1
		2.5E-02	LSEC 3
hsa04066	HIF-1 Signaling Pathway	6.4E-03	Cholangiocyte 1
		2.3E-03	Cholangiocyte 2
		2.5E-03	Macrophage 1

		1.9E-03	Macrophage 2
		4.2E-03	Macrophage 3
		2.6E-04	Hepatocyte 1
		8.1E-03	Hepatocyte 2
		2.3E-03	Hepatocyte 4
		2.3E-04	Hepatocyte 5
		2.7E-04	Hepatocyte 6
		7.0E-03	Hepatocyte 7
		2.8E-03	Hepatocyte 8
		1.8E-04	Hepatocyte 9
		6.3E-03	Hepatocyte 11
		2.3E-03	HSC 1
		2.1E-02	HSC 4
		2.7E-05	LSEC 1
		2.4E-03	LSEC 2
		4.6E-02	LSEC 3
		1.1E-02	LSEC 4

Abbreviations: BMPR2 – Bone morphogenic Protein Receptor Type 2; ESR1 – Estrogen Receptor 1; FLT1 – FMS-like tyrosine kinase 1 (Vascular endothelial growth factor receptor 1); VEGF – Vascular Endothelial Growth Factor; HIF-1 – Hypoxia-inducible Factor 1; HSC – Hepatic Stellate Cell; FDR – False Discovery Rate

Female only refers to samples from subjects 479, 566, 397, 455, 448, and 557

Supplementary Table 2: Differential Expression of Inflammatory Cytokine and Chemokine Genes Across Clusters

Differential Gene Expression Across Clusters			
Gene	Average Log-2 Fold Change Between Groups	FDR-Adjusted P-value	Cluster
INFGR1	-0.362	6.1E-03	Hepatocyte 10
	-0.315	1.3E-02	Hepatocyte 5
IL7	-0.291	1.1E-02	HSC 3
IL7R	-0.917	9.6E-42	Hepatocyte 2
IL6R	0.803	1.2E-85	Hepatocyte 3
	0.381	3.4E-31	Hepatocyte 4
	0.424	1.0E-46	Hepatocyte 7
	0.393	4.1E-33	Hepatocyte 8
	0.321	1.5E-26	Hepatocyte 9
	0.268	1.7E-08	Hepatocyte 11
	0.494	2.3E-07	LSEC 4
	0.335	2.0E-02	HSC S1
	0.351	9.6E-05	Cholangiocyte 4
IL4R	0.452	7.6E-22	Hepatocyte 1
	0.573	1.4E-40	Hepatocyte 3
	0.512	1.9E-51	Hepatocyte 4
	0.372	1.4E-30	Hepatocyte 6
	0.463	5.0E-32	Hepatocyte 7
	0.356	9.2E-27	Hepatocyte 8
	0.379	2.0E-33	Hepatocyte 9
	0.391	7.9E-19	Hepatocyte 11
	0.706	8.0E-04	Macrophage 4
	0.372	3.4E-05	LSEC 4
	0.355	1.8E-04	LSEC 1
IL32	-0.686	7.4E-61	Hepatocyte 4
	-0.456	2.4E-31	Hepatocyte 5
	-0.464	2.5E-26	Hepatocyte 6
	-0.402	1.9E-19	Hepatocyte 7
	-0.332	1.4E-15	Hepatoyte 8
	-0.418	3.3E-15	Hepatocyte 9
	-0.396	6.1E-11	Hepatocyte 10
	-0.410	9.0E-20	Hepatocyte 11
	-0.599	2.7E-15	Macrophage 1
	-0.573	5.3E-16	Macrophage 2
	-0.765	7.1E-17	Macrophage 3
	-0.659	6.1E-22	LSEC 1
	-0.446	1.7E-06	LSEC 3
	-0.442	2.0E-02	LSEC 2
	-0.404	3.7E-02	HSC S1

	-0.397	4.0E-02	LSEC 4
	-0.447	1.0E-02	Cholangiocyte 3
	-0.998	4.1E-109	Hepatocyte 1
	-0.496	1.0E-18	Hepatocyte 2
	-0.733	2.5E-55	Hepatocyte 3
	-0.573	5.3E-16	Macrophage 2
IL34	-0.517	1.5E-07	HSC3
	-0.388	9.8E-06	HSC2
	-0.324	5.4E-05	HSC1
IL2RA	0.405	6.0E-03	Macrophage 3
MIF	-0.345	8.0E-03	Macrophage 1
	-0.335	1.6E-03	Macrophage 2
	-0.347	1.6E-02	Endothelial
	-0.341	5.5E-05	LSEC 1
	-0.599	5.7E-39	Hepatocyte 1
	-0.286	7.0E-04	Hepatocyte 2
	-0.523	6.6E-29	Hepatocyte 3
	-0.386	6.9E-19	Hepatocyte 4
CXCL2	0.789	1.3E-13	Cholangiocyte 4
	0.361	6.0E-05	Hepatocyte 2
	0.948	9.7E-100	Hepatocyte 3
	0.657	1.6E-59	Hepatocyte 4
	0.274	2.3E-09	Hepatocyte 5
	0.361	6.5E-16	Hepatocyte 6
	0.605	6.5E-43	Hepatocyte 7
	0.594	6.7E-46	Hepatocyte 8
	0.383	2.8E-12	Hepatocyte 9
	0.259	2.0E-03	Hepatoyte 10
	0.607	6.9E-37	Hepatocyte 11
	0.792	5.7E-09	Hepatocyte S1
	0.654	7.4E-13	Macrophage 1
	0.505	1.4E-07	Macrophage 2
	1.292	2.5E-12	Macrophge 4
	1.023	1.1E-26	LSEC 4
	0.678	2.4E-23	LSEC 1
	0.838	5.2E-14	HSC S1
CD163	0.681	2.1E-56	Hepatocyte 1
	0.364	2.5E-05	Hepatocyte 2
	0.526	1.4E-25	Macrophage 2
	0.421	1.7E-09	Macrophage 3
	0.457	8.7E-03	HSC 3

Abbreviations: INFGR1 – Interferon Gamma Receptor 1; IL7 – Interleukin 7; IL7R – Interleukin 7 Receptor; IL6R – Interleukin 6 Receptor, IL4R – Interleukin 4 Receptor, IL32 – Interleukin 32; IL34 – Interleukin 34, IL2RA – Interleukin 2 Receptor Alpha Subunit; MIF – Macrophage

Migration Inhibitory Factor; CXCL2 – Chemokine (C-X-C motif) Ligand 2; HSC – Hepatic Stellate Cell; LSEC – Liver Sinusoidal Endothelial Cell; FDR – False Discovery Rate

Supplementary Table 3: Log-Transformed Semi-Quantitative Immunohistochemistry Data

Protein	PoPH	Non-PoPH Cirrhosis	Non-Cirrhosis Healthy Liver
	Median (IQR)	Median (IQR)	Median (IQR)
Central Vein ESR1	-7.2 (-7.5, -6.2)	-9.4 (-9.6, -9.4)	-9.6 (-10.6, -8.5)
Central Vein BMPR2	-6.8 (-6.9, -5.9)	-4.2 (-4.6, -4.1)	-6.2 (-7.3, -5.2)
Central Vein FLT1	-7.2 (-7.3, -6.2)	-8.5 (-9.2, -8.3)	-6.7 (-9.0, -5.9)
Portal Triad ESR1	-6.0 (-7.4, -4.8)	-7.1 (-8.2, -5.7)	-5.6 (-6.1, -5.1)
Portal Triad BMPR2	-3.8 (-3.9, -3.8)	-3.7 (-3.2, -3.1)	-3.5 (-3.7, -3.3)
Portal Triad FLT1	-6.0 (-6.8, -5.2)	-5.4 (-6.4, -5.3)	-6.0 (-7.3, -5.5)

Abbreviations: PoPH – Portopulmonary Hypertension, IQR – Inter-Quartile Range

Supplementary Table 4: Relationship Between Biomarkers and PoPH Hemodynamic Disease Severity

Biomarker Initial Discovery Cohort	mPAP (mmHg)		PVR (Wood units)		CI (L/min/m^2)	
	Correlation Tau	p-value	Correlation Tau	p-value	Correlation Tau	p-value
Plasma FLT1 (pg/ml)	0.107	0.166	0.056	0.464	0.000	>0.999
Serum NTproBNP (pg/ml)	0.046	0.541	0.026	0.732	-0.038	0.626
Plasma BMP9 (pg/ml)	-0.037	0.630	0.038	0.618	-0.133	0.089
Biomarker Second Validation Cohort	mPAP (mmHg)		PVR (Wood units)		CI (L/min/m^2)	
	Correlation Tau	p-value	Correlation Tau	p-value	Correlation Tau	p-value
Plasma FLT1 (pg/ml)	-0.040	0.785	-0.051	0.750	0.022	0.881
Serum NTproBNP (pg/ml)	-0.294	0.152	0.056	0.784	-0.162	0.435

Abbreviations: PoPH – Portopulmonary Hypertension, mPAP – Mean Pulmonary Arterial Pressure, PVR – Pulmonary Vascular Resistance, CI – Cardiac Index, FLT1 – FMS Related Receptor Tyrosine Kinase 1, BMP9 – Bone Morphogenic Protein Type 9, NTproBNP – N-Terminal prohormone of Brain natriuretic Peptide

Supplementary Table 5: Sequencing Quality Control Summaries

Sample	458	452	479	566	593	397	455	448	557
Disease	EtOH /HCV Cirrhosis	EtOH Cirrhosis	HCV Cirrhosis	PBC Cirrhosis	EtOH Cirrhosis	NASH Cirrhosis PoPH	HCV Cirrhosis PoPH	EtOH Cirrhosis PoPH	EtOH Cirrhosis PoPH
Age	60	48	69	67	34	55	71	64	42
Sex	M	M	F	F	M	F	F	F	F
Ethnicity	Black	Black	White	White	White	White	White	White	White
MELD	10	36	7	40	25	11	15	7	7
Total Reads	422,510,709	439,146,728	436,535,189	497,859,374	361,867,514	423,062,765	488,593,228	373,847,150	477,211,489
Sequencing saturation	48.1%	27.3%	33.4%	33.4%	27.0%	42.4%	77.6%	25.9%	33.5%
Mapped to genome	95.0%	92.4%	92.3%	86.7%	90.9%	94.6%	96.2%	86.9%	89.5%
Estimated Cells	8701	9960	9,875	11,718	10,632	7,689	5,863	10,488	12,332
Mean reads per cell	48,559	44,091	44,206	42,487	34,036	55,022	83,335	35,645	38,697
Median genes per cell	2,486	2,191	2,262	2,026	2,059	2,446	2,289	1,226	2,169
Median UMI per cell	4,416	3,486	3,874	3,301	3,119	4,393	4,884	1,680	3,766

Supplementary Table 6: Missing Samples for Biomarker Testing from Biorepository Cohorts

Biomarker	Initial Validation Cohort	Missing (N, %)
Serum NTproBNP	Non-PoPH Cirrhosis	6 (12%)
Plasma FLT1/VEGFR1	Non-PoPH Cirrhosis<br>PoPH	20 (33%)<br>2 (3%)
Biomarker	Second Independent Cohort	Missing (N, %)
Plasma FLT1/VEGFR1	Non-PoPH Cirrhosis<br>PoPH	1 (1%)<br>1 (4%)

PVCLD2 Consortium Members:

Additional members of the Pulmonary Vascular Complications of Liver Disease Study Group are: Cleveland Clinic Foundation: Gustavo A. Heresi, MD, Raed A. Dweik, MD, Kasi Timmerman, GED; Mayo Clinic: Jae K. Oh, MD, Grace Lin, MD, C.D. Mottram, RRT, Paul D. Scanlon, MD, Adam Miller, BA; Penn State University: Karen L. Krok MD; Tufts Medical Center: Karen Visnaw, RN; University of Edinburgh: Natalie Homer, SD, University of Colorado: Todd M. Bull, MD, Cheryl Abbott, RN; University of Pennsylvania School of Medicine: Nadine Al-Naamani, MD, Kimberly A. Forde, MD, PhD, MHS, Mamta J. Patel, RN, Harold I. Palevsky, MD, K. Rajender Reddy, MD, David S. Goldberg, MD, MSCE, Vandana Khungar, MD, MSc, K. Akaya Smith, MD, Jason S. Fritz, MD, Marita Lynch, BA, Tiffany Sharkoski, MPH, Diane Pinder, BSc; University of Texas–Houston: Victor Machicao, MD, Moises Nevah Rubin, MD, Kim Walker, CNA, Stacy Cranford, RN, Jordan Varing, MPH; University of Texas–Southwestern: Sonja Bartolome, MD, Namrata Banga, MD, CCRP, RAC, Oluwatosin Igenoza, MD, MPH; Vanderbilt University: Eric D. Austin, MD, MS, Anna R. Hemnes, MD, Celeste LaRochelle.
