# Supplementary material for: Human liver single nuclear RNA sequencing implicates BMPR2, GDF15, arginine, and estrogen in portopulmonary hypertension
Source: Commun Biol. 2023 Aug 9;6:826. doi: 10.1038/s42003-023-05193-3 (PMC10412637; doi:10.1038/s42003-023-05193-3)
Supplement: Supplementary file 2 — Description of Additional Supplementary Files [file 42003_2023_5193_MOESM2_ESM.pdf]

Description of Additional Supplementary Files

File name: Supplementary Data 1
Description: Marker Gene Expression Data, the source data for Fig. 1A-1C and Supplementary Fig. 1 and 2.

File name: Supplementary Data 2
Description: Differentially Expressed Gene Data, the source data for Fig. 2, Fig. 3A-3B, Supplementary Figure 3, 4, 5, 8, 9, 10, and 11 and Supplementary Tables 1 and 2

File name: Supplementary Data 3
Description: Biomarker and Immunohistochemistry Data, the source data for Fig. 3C-3E, Supplementary Fig. 7, and Tables 2 and 3, and Supplementary Tables 3, 4, and 6
