# Supplementary material for: Human liver single nuclear RNA sequencing implicates BMPR2, GDF15, arginine, and estrogen in portopulmonary hypertension
Source: Commun Biol. 2023 Aug 9;6:826. doi: 10.1038/s42003-023-05193-3 (PMC10412637; doi:10.1038/s42003-023-05193-3)
Supplement: Supplementary file 4 — Reporting Summary [file 42003_2023_5193_MOESM4_ESM.pdf]

Corresponding author(s): Arun Jose MD MS
Last updated by author(s): Jul 19, 2023

Reporting Summary

Nature Portfolio wishes to improve the reproducibility of the work that we publish. This form provides structure for consistency and transparency in reporting. For further information on Nature Portfolio policies, see our Editorial Policies and the Editorial Policy Checklist.

Statistics

For all statistical analyses, confirm that the following items are present in the figure legend, table legend, main text, or Methods section.

n/a	Confirmed	
☐	☒	The exact sample size (*n*) for each experimental group/condition, given as a discrete number and unit of measurement
☐	☒	A statement on whether measurements were taken from distinct samples or whether the same sample was measured repeatedly
☐	☒	The statistical test(s) used AND whether they are one- or two-sided<br>*Only common tests should be described solely by name; describe more complex techniques in the Methods section.*
☐	☒	A description of all covariates tested
☐	☒	A description of any assumptions or corrections, such as tests of normality and adjustment for multiple comparisons
☐	☒	A full description of the statistical parameters including central tendency (e.g. means) or other basic estimates (e.g. regression coefficient) AND variation (e.g. standard deviation) or associated estimates of uncertainty (e.g. confidence intervals)
☐	☒	For null hypothesis testing, the test statistic (e.g. *F*, *t*, *r*) with confidence intervals, effect sizes, degrees of freedom and *P* value noted<br>*Give P values as exact values whenever suitable.*
☒	☐	For Bayesian analysis, information on the choice of priors and Markov chain Monte Carlo settings
☒	☐	For hierarchical and complex designs, identification of the appropriate level for tests and full reporting of outcomes
☐	☒	Estimates of effect sizes (e.g. Cohen's *d*, Pearson's *r*), indicating how they were calculated

Our web collection on statistics for biologists contains articles on many of the points above.

Software and code

Policy information about availability of computer code

Data collection	No software was used
Data analysis	Seurat package version 4.0.6, the scrublet package, the CellChat package, the Fiji ImageJ2 platform version 2.9.0/1.53t, and R version 4.1.2 were used for data analysis

For manuscripts utilizing custom algorithms or software that are central to the research but not yet described in published literature, software must be made available to editors and reviewers. We strongly encourage code deposition in a community repository (e.g. GitHub). See the Nature Portfolio guidelines for submitting code & software for further information.

Data

Policy information about availability of data

All manuscripts must include a data availability statement. This statement should provide the following information, where applicable:

- Accession codes, unique identifiers, or web links for publicly available datasets
- A description of any restrictions on data availability
- For clinical datasets or third party data, please ensure that the statement adheres to our policy

All single nucleus RNA sequencing data generated by this study have been deposited in the Mendeley Data archive (Jose, Arun (2023), “Human single nuclear RNA seq PoPH and non-PoPH Cirrhosis Liver”, Mendeley Data, V1, doi: 10.17632/y2s67z8tw9.1).

Human research participants

Policy information about studies involving human research participants and Sex and Gender in Research.

Reporting on sex and gender	Sex/Gender were self-reported. In the samples of liver tissue for snRNAseq, subjects were predominantly female sex (67%), and IHC tissue samples were 55% female (Table 1). Sex was also self-reported for the initial and secondary biomarker validation cohort (Table 2), with 44% female in initial validation cohort, and 39% in second validation cohort female sex. Sex was reported in the healthy non-diseased samples (83% Female, Extended Data Table 4). Sex was used as a covariate for multivariable regression analysis when considering ability of candidate biomarkers to discriminate PoPH from other disease subtypes.
Population characteristics	For the snRNAseq tissue, subjects were between the ages of 34 and 71, majority white race (89%), and 4 were PoPH on targeted therapy at time of liver transplant, 5 were non-PoPH cirrhosis of different liver disease etiologies (EtOH, HCV, PBC cirrhosis). Initial and secondary validation cohorts characteristics were as follows: For initial cohort, median age was 58 years, majority white race, and most of the non-PoPH cirrhosis had HCV, with a lower proportion of EtOH and NASH cirrhosis. Treatment information on the PoPH subjects in this cohort, and the etiology of liver disease in the PoPH subjects in this cohort, was not available. For the secondary validation cohort, median age 58, majority white race, and most had EtOH cirrhosis, with lower proportions of HCV, NASH, and PBC Cirrhosis.
Recruitment	Patients for the snRNAseq analysis were recruited in clinic at the time of evaluation for PoPH awaiting liver transplant. There are selection biases present, as only those subjects able to undergo liver transplant would be included in the study. Additionally, tissue samples occur at time of liver transplant (later-stage disease), and study results may not be generalizable to earlier-stage PoPH. The biomarker samples were obtained from repositories, and patients were recruited and enrolled at time of evaluation in pulmonary hypertension or liver disease clinic. The initial validate cohort did not have information on PoPH patient liver disease etiology or treatment with targeted PAH therapy. Both cohorts were selected randomly from the full biorepositories.
Ethics oversight	Samples were collected with institutional ethics approval from the University of Cincinnati IRB (UC IRB 2013-8157, UC IRB 2012-3689).

Note that full information on the approval of the study protocol must also be provided in the manuscript.

Field-specific reporting

Please select the one below that is the best fit for your research. If you are not sure, read the appropriate sections before making your selection.

☒ Life sciences ☐ Behavioural & social sciences ☐ Ecological, evolutionary & environmental sciences

For a reference copy of the document with all sections, see nature.com/documents/nr-reporting-summary-flat.pdf

Life sciences study design

All studies must disclose on these points even when the disclosure is negative.

Sample size	No sample size was performed. The liver tissue used for snRNAseq was a sample of convenience, and this technique has not been applied to human liver with PoPH before, and is similar to other reports of single cell and snRNAseq in human liver tissue (Macparland S et al. Nat Commun 22:4383 (2018); Andrews TS, et al. Hepatol Commun 6: 821-840 (2022)), so we felt this sample size was sufficient. We did not perform sample size calculation for the initial validation and second independent validation cohorts of biomarkers, instead using the majority of PoPH samples available from both cohorts. We believe this sample size is sufficient as it is larger than several other studies of published PoPH biomarkers (Al-Naamani N et al. Hepatology 73, 726-737 (2021); Nikolic I et al. Am J Respir Crit Care Med 199, 891-902 (2019); Ronchon ER et al. Am J Respir Crit Care Med 201, 1575-1578 (2020); Owen NE et al. EBioMedicine 56, 102794 (2020)).
Data exclusions	No data excluded from the analyses
Replication	We replicated the ESR1 and BMPR2 gene expression patterns of PoPH liver tissue seen on snRNAseq using immunohistochemistry studies of human liver tissue. We validated biomarker results from the initial validation cohort (from the National PAH Biobank) in a second independent validation cohort using the PVCLD2 cohort.
Randomization	Allocation was not random. Analyses of biomarker relationships with disease etiology (PoPH or non-PoPH cirrhosis) were performed using logistic regression models. Biomarker levels were log-transformed prior to logistic regression analysis. Significant univariate regression models were adjusted in a multivariable fashion for clinical covariates (age, sex, race, and body mass index (BMI)) in the initial validation cohort comprised of PoPH, non-PoPH cirrhosis, and IPAH subjects.
Blinding	Blinding was not relevant, the identify of subject samples (PoPH, non-PoPH Cirrhoisis, IPAH) was necessary for analysis and interpretation.

Reporting for specific materials, systems and methods

We require information from authors about some types of materials, experimental systems and methods used in many studies. Here, indicate whether each material, system or method listed is relevant to your study. If you are not sure if a list item applies to your research, read the appropriate section before selecting a response.

Materials & experimental systems

n/a	Involved in the study
☐	☒ Antibodies
☒	☐ Eukaryotic cell lines
☒	☐ Palaeontology and archaeology
☒	☐ Animals and other organisms
☒	☐ Clinical data
☒	☐ Dual use research of concern

Methods

n/a	Involved in the study
☒	☐ ChIP-seq
☒	☐ Flow cytometry
☒	☐ MRI-based neuroimaging

Antibodies

Antibodies used	Immunohistochemical study of the expression levels of molecular markers FLT1 (VEGFR1), BMPR2, and the estrogen receptor ESR1 was carried out using the following primary antibodies: (i) recombinant rabbit anti-VEGF Receptor 1 monoclonal IgG antibody (ab32152, Abcam, Great Britain), (ii) recombinant rabbit anti-Estrogen Receptor alpha monoclonal IgG antibody (ab108398, Abcam, Great Britain), (iii) recombinant rabbit anti-BMPR2 polyclonal IgG antibody (ab124463, Abcam, Great Britain); and secondary antibody: (iv) unconjugated polyclonal rabbit anti-goat IgG (H+L) secondary antibody (PISA510312, Fisher Scientific, Vilnius, Lithuania). Tissue samples were first deparaffinized by washing in distilled water, followed by antigen retrieval using a citrate buffer solution. Sections were then blocked first with BLOXALL endogenous peroxide blocker (Vector Laboratories BLOXALL endogenous peroxide and alkaline phosphatase blocking solution 100ml, NC0185217, Fisher Scientific, Vilnius, Lithuania). Primary staining (FLT1 antibody at a dilution of 1:200, BMPR2 antibody at a dilution of 1:75, and ESR1 antibody at a dilution of 1:250) was applied overnight, followed by secondary staining using the rabbit anti-goat antibody listed above at a dilution of 1:500. Slides were then developed using DAPI (Vector Laboratories DAPI, Fisher Scientific, Vilnius, Lithuania), countered with hematoxylin, and dehydrated.
Validation	Antibodies used for immunohistochemistry served as a validation for the single nuclear RNA sequencing data
